# Alleviation of C⋅C Mismatches in DNA by the *Escherichia coli* Fpg Protein

**DOI:** 10.3389/fmicb.2021.608839

**Published:** 2021-06-30

**Authors:** Almaz Nigatu Tesfahun, Marina Alexeeva, Miglė Tomkuvienė, Aysha Arshad, Prashanna Guragain, Arne Klungland, Saulius Klimašauskas, Peter Ruoff, Svein Bjelland

**Affiliations:** ^1^Department of Chemistry, Bioscience and Environmental Technology, Faculty of Science and Technology, University of Stavanger, Stavanger, Norway; ^2^Department of Biological DNA Modification, Institute of Biotechnology, Vilnius University, Vilnius, Lithuania; ^3^Department of Microbiology, Oslo University Hospital, Oslo, Norway; ^4^Department of Molecular Medicine, Life Sciences Center, Institute of Basic Medical Sciences, University of Oslo, Oslo, Norway; ^5^Department of Clinical Molecular Biology, Akershus University Hospital, Lørenskog, Norway

**Keywords:** DNA base mismatch, cytosine:cytosine mismatch, thymine:thymine mismatch, base excision repair, DNA glycosylase, *Escherichia coli* Fpg, mutM

## Abstract

DNA polymerase III mis-insertion may, where not corrected by its 3′→ 5′ exonuclease or the mismatch repair (MMR) function, result in all possible non-cognate base pairs in DNA generating base substitutions. The most thermodynamically unstable base pair, the cytosine (C)⋅C mismatch, destabilizes adjacent base pairs, is resistant to correction by MMR in *Escherichia coli*, and its repair mechanism remains elusive. We present here *in vitro* evidence that C⋅C mismatch can be processed by base excision repair initiated by the *E. coli* formamidopyrimidine-DNA glycosylase (Fpg) protein. The *k*_cat_ for C⋅C is, however, 2.5 to 10 times lower than for its primary substrate 8-oxoguanine (oxo^8^G)⋅C, but approaches those for 5,6-dihydrothymine (dHT)⋅C and thymine glycol (Tg)⋅C. The *K*_M_ values are all in the same range, which indicates efficient recognition of C⋅C mismatches in DNA. Fpg activity was also exhibited for the thymine (T)⋅T mismatch and for *N*^4^- and/or 5-methylated C opposite C or T, Fpg activity being enabled on a broad spectrum of DNA lesions and mismatches by the flexibility of the active site loop. We hypothesize that Fpg plays a role in resolving C⋅C in particular, but also other pyrimidine⋅pyrimidine mismatches, which increases survival at the cost of some mutagenesis.

## Introduction

All possible base mismatches in DNA are formed in *Escherichia coli* by the replicative DNA polymerase (Pol) III holoenzyme. *E. coli* also contains a few trans-lesion synthesis (TLS) Pols that can insert cognate or non-cognate bases opposite damaged and undamaged template bases, including at apurinic/apyrimidinic (AP) sites. This can result in a cytosine (C)⋅C mismatch. A number of studies suggest that Pol III generally leaves the processivity (β) clamp at the replication fork, if a mispair such as C⋅C evades the Pol III 3′→ 5′ proofreading exonuclease (exo) function. This can be replaced by the TLS Pol IV (DinB), which is able to continue synthesis from the mismatch to a much greater extent than Pol III (Wagner and Nohmi, 2000). Despite the *dinB* gene being SOS-induced, the substantial number of molecules of Pol IV per cell (150–250) under normal conditions (Fijalkowska et al., 2012) suggest that SOS induction is not necessary for the generation of the C⋅C mismatch. The C⋅C mismatch is, unlike all other mismatches, resistant to correction by the *E. coli* MutHLS mismatch repair (MMR) system (Iyer et al., 2006), a G⋅C → C⋅G mutation being the obvious consequence of this. There is, therefore, a lack of evidence of how C⋅C mismatch mutation avoidance occurs. Other C⋅C MMR mechanisms have yet to be identified. The C⋅C mismatch might also be a challenge to genomic integrity under conditions of slow DNA synthesis or replicative arrest. The C⋅C pair is the most thermodynamically unstable mismatch and can therefore destabilize 7–9 adjacent base pairs (Tikhomirova et al., 2006), and so be a target for chemical and endonuclease attack and the generation of double strand (ds) breaks in DNA (Friedberg et al., 2006). This contrasts the guanine (G)⋅G, adenine (A)⋅A, and thymine (T)⋅T mismatches, only the two adjacent base pairs being affected (Tikhomirova et al., 2006). It was, interestingly, reported some years ago that the *E. coli* formamidopyrimidine-DNA glycosylase [Fpg (Boiteux et al., 2017); also known as MutM] binds to the C⋅C mismatch *in vitro*. No enzyme activity was, however, detected. It was therefore concluded that Fpg may recruit other components to perform the repair reactions (Nakahara et al., 2000).

The Nakahara et al. report is compounded by our recent discovery that the *E. coli* Fpg exhibits significant activity where *N*^4^,5-dimethylcytosine (m*^*N*^*^4,5^C) placed opposite C or T is removed from DNA *in vitro* (Alexeeva et al., 2018). The report and our discovery urged us to investigate in detail the ability of Fpg, and therefore the base excision repair (BER) pathway, to incise and repair C, including where methylated C is opposite C. We found that the enzyme exhibited activity for C⋅C. This includes where *N*^4^-methylcytosine (m*^*N*^*^4^C) and/or 5-methylcytosine (m^5^C) are opposite both C and T. Fpg also incises the T⋅T homo-mismatch with a level of efficiency that is similar to C⋅C.

## Materials and Methods

### Oligonucleotide Substrates

**FIGURE 1:**
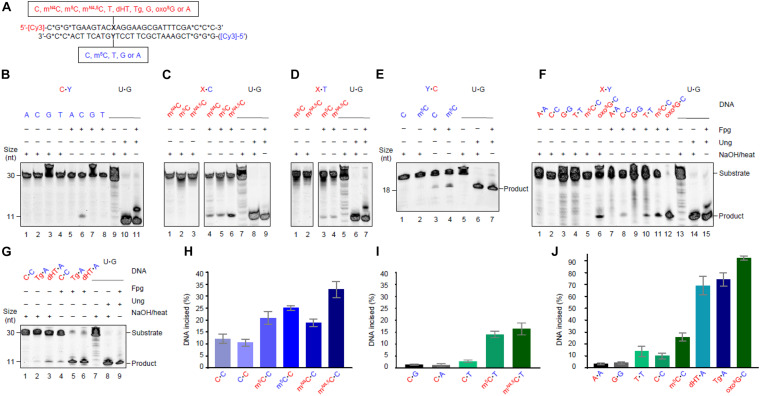
*Escherichia coli* Fpg protein incises at unmethylated and methylated cytosine when placed opposite C and T in DNA. **(A)** Schematic representation of DNA substrates. Fluorescently labeled DNA oligonucleotides (*, phosphodiester bonds protected by phosphorothioate) had a spectrum of different studied bases at one site, as indicated. The upper strand was primarily labeled, its variable base defined by *X* (indicated in red); the lower strand was occasionally labeled, its variable base defined by *Y* (indicated in blue). The color code is kept throughout all the subsequent figures and tables indicating which variables in which strand were tested in a particular experiment. A variable base in the labeled strand (the assessed one) is always written as the first in a base pair. See Supplementary Figure 1 for an outline of the assay. **(B)** Activity for C opposite all major bases. **(C)** Activity for methylated Cs opposite C. **(D)** Activity for methylated Cs opposite T. In panels **(B–D)**, DNA (upper strand labeled) X substrate [see panel **(A)**, 1 pmol] was incubated alone (lanes 1–4) or with Fpg (13 pmol; lanes 5–8) at 37°C in NEB1 buffer (10 mM Bis–Tris-propane-HCl, pH 7.0, 10 mM MgCl_2_), 1 mM DTT, 0.1 mg/mL BSA for 1 h. U⋅G-DNA (30 nt; 1 pmol) was incubated without (lane 9) or with Ung (1.95 pmol; lane 10) followed by NaOH/heat treatment, and was used as a negative and positive control, respectively, for active Ung, and to convert U⋅G-DNA into AP-DNA to demonstrate active Fpg (i.e., AP lyase activity; lane 11). **(E)** Activity for C and m^5^C opposite C, when the lower strand with the *Y* variable was labeled. DNA substrate (1 pmol) was incubated alone (lanes 1 and 2) or with Fpg (lanes 3 and 4) using the controls (lanes 5–7) described above. **(F)** Activity for C, m^5^C, or oxo^8^G opposite C compared to the other homo-mismatches. **(G)** Activity for C opposite C compared to Tg and dHT opposite A. In panels **(F,G)**, DNA (upper strand labeled) X substrate [see panel **(A)**, 1 pmol] was incubated alone (lanes 1–6 and 1–3, respectively) or with Fpg (lanes 7–12 and 4–6, respectively) using the controls (lanes 13–15 and 7–9, respectively) described above. **(H)** The percent of the labeled strand incised at unmethylated and methylated C opposite C. **(I)** The percent of the labeled strand incised at unmethylated C opposite T, A, or G compared to methylated C opposite T. **(J)** The percent of the labeled strand incised at most homo-mismatches compared to oxidized bases in DNA. These column graphs show the average values (±SD) obtained from 4 to 10 independent experiments as presented in panels **(B–G)**, the first base in each pair being the one assayed.

Equimolar amounts of single-stranded (ss) forward (Fw) [Cy3] 5′-C^∗^G^∗^G^∗^TGAAGTAC[X]AGGAAGCGATTTCGA^∗^C^∗^C^∗^C-3′ (X = C, m*^*N*^*^4^C, m^5^C, m*^*N*^*^4,5^C, T, 5,6-dihydrothymine (dHT), thymine glycol (Tg), G, 8-oxoguanine (oxo8G) or A; fluorescently labeled with Cy3 from Sigma-Aldrich) and reverse (Rev) 5′-G^∗^G^∗^G^∗^TCGAAATCGCTTCCT[Y]GTACTTCA^∗^C^∗^C^∗^G-3′ (Y = G, m^5^C, A, or T) polydeoxyribonucleotides end-protected by phosphorothioates (^∗^) were annealed to form a DNA duplex of 30 nucleotides (nt). This resulted in an 11-nt incision product with an active glycosylase enzyme (see Figure 1A). The DNA at the defined site, which contains m*^*N*^*^4^C and m*^*N*^*^4,5^C, was prepared as described previously by Alexeeva et al. (2018). The ss polydeoxyribonucleotide [Cy3] 5′-CCCTCGAT GTA[U]CATGGATCCGATCGATCC-3′ (Fw 30 nt; 11 nt incision product) containing uracil (U) at the specific site was annealed to equimolar amounts of the Rev strand with G opposite U, and used as a positive control substrate [for active enzyme, Fpg AP lyase activity, after incubation with uracil-DNA glycosylase (Ung)]. The ss polydeoxyribonucleotides (from Sigma-Aldrich) [Cy3] 5′-TAGACATTGCCCTCGAGGTATCATGGATCCGATTTCGAC [C]TCAAACCTAGACGAATTCCG-3′ (Fw 60 nt; 39 nt incision product) were, in the experiments that include non-denaturing polyacrylamide gel electrophoresis (PAGE) (see Supplementary Figure 4), annealed to equimolar amounts of the Rev strand with C opposite C. The corresponding Fw control U oligonucleotide [Cy3] 5′-TAGACATTGCCCTCGAGGTATCATGGATCCGATTTCGAC [U]TCAAACCTAGACGAATTCCG-3′ (Fw 60 nt; 39 nt incision product) was annealed to equimolar amounts of the Rev strand with G opposite U.

### Repair Enzymes

Formamidopyrimidine-DNA glycosylase [Cat. No. M0240S; 8,000 U/mL (13 pmol/μL); lot No. 0061405; dissolved in 20 mM Tris–HCl, pH 8.0, 50 mM NaCl, 0.5 mM ethylenediaminetetraacetic acid (EDTA), 200 μg/mL BSA, 50% glycerol], Ung [Cat. No. M0280S; 5,000 U/mL (1.95 pmol/μL)], Nfo [Cat. No. M0304S; 10,000 U/mL (83 nM)], and polynucleotide kinase (PseT) [Cat. No. M0201S; 10,000 U/mL (0.29 μM)] were obtained from New England Biolabs. Fpg preparation was subjected to mass-spectrometric (MS) analysis, so ruling out contaminating activity (Supplementary Table 1).

### DNA Excision and Incision Assay

Purified DNA glycosylase (Fpg and/or Ung) was, if not otherwise stated, incubated with substrate DNA (see Figure 1A) at 37°C in 10 mM Bis–Tris-propane-HCl, pH 7.0, 10 mM MgCl_2_, 1 mM dithiothreitol (DTT), 0.1 mg/mL BSA (final volume, 20 μL). Reactions were terminated by the addition of 20 mM EDTA, 0.5% (w/v) sodium dodecyl sulphate (SDS), and proteinase K (150 μg/mL) and incubated at 37°C for 10 min. DNA was precipitated with ethanol and the precipitate was solubilized in water (10 μL if not otherwise stated) (Leiros et al., 2007). DNA glycosylase activity was determined by a NaOH-mediated (0.1 M final concentration) incision of the resulting AP site (90°C for 10 min), incision of the DNA without alkaline treatment demonstrating enzymatic DNA incision activity (see Supplementary Figure 1). A loading solution containing 80% (v/v) formamide, 1 mM EDTA, and 1% (w/v) blue dextran (10 μL) was added and the mixture was incubated at 95°C for 5 min to denature DNA. The vial was then cooled on ice, and then centrifuged at 4°C for a short period of time. The samples (5 μL) were subjected to PAGE using a gel [20% (w/v) polyacrylamide (acrylamide:bis-acrylamide 37.5:1)] containing 8 M urea. PAGE was performed using a Tris-borate-EDTA buffer system (89 mM Tris base, 89 mM boric acid, 2 mM EDTA, pH 8.0), usually at 200 V for 2 h. Visualization and quantification were performed using fluorescence imaging analysis and ImageQuant Software (Molecular Dynamics Inc.). This procedure was also used for non-denaturing PAGE. A non-denaturing loading solution containing 10 mM Tris, pH 7.6, 60% (v/v) glycerol, 60 mM EDTA, and 1% (w/v) blue dextran (10 μL) was, however, used after DNA precipitation and solubilization in water.

### Trapping Experiment for Schiff Base Intermediate

The assay was performed following the described method (Zharkov et al., 1997). Fluorescently 5′-labeled dsDNA (1 pmol, see Figure 1A; obtained from Sigma-Aldrich, not end-protected) was incubated with Fpg (10 pmol) in 45 mM HEPES [4-(2-hydroxyethyl)-1-piperazineethanesulphonic acid]-KOH, pH 7.5, 2% (v/v) glycerol, 0.4 mM EDTA, 1 mM DTT (final volume, 10 μL), and in the presence of freshly dissolved 50 mM sodium borohydride, for 1 h at 37°C. An oligomer containing uracil was, for uracil residue excision, incubated with both *E. coli* Ung and Fpg (10 pmol each) as a positive control. Reactions were stopped after sodium borohydride reduction by adding an equal volume of denaturing loading buffer containing 80% (v/v) formamide, 1 mM EDTA, and 0.05% (w/v) bromophenol blue. Samples were furthermore denatured for 5 min at 95°C and separated on 10% (w/v) denaturing PAGE at 200 V for 54 min.

### Kinetic and Computational Methods

#### Irreversible Michaelis–Menten Equation

The rate equations of the irreversible Michaelis–Menten mechanism

(1) $$\text{E}+\text{S}\overset{k_{1}}{\underset{k_{-1}}{⇄}}\text{ES}\overset{k_{2}}{\to }\text{E}+\text{P}$$

were analyzed numerically using the FORTRAN subroutine LSODE (Radhakrishnan and Hindmarsh, 1993). The numerical solutions were compared by using the rapid equilibrium approximation between E, S, and the enzyme-substrate complex ES, and by the steady-state approximation where the time derivative of [ES] is 0 (Segel, 1975). The validity to use the steady state approach of the Michaelis–Menten equation is given in detail in the Section “Validity of the Michaelis–Menten Approach” in the Supplementary Material. In addition, implied pseudo-first-order kinetics are indeed observed when compared with the kinetic data (see Supplementary Tables 3, 4).

#### Determination of Reaction Velocity

The “initial velocity” *v*_0_ was determined by quantifying the amount of formed product P after 30 min incubation using fluorescence image analysis (see section “DNA Excision and Incision Assay” above).

#### Determination of Rate Parameters

The rate parameters *V*_max_, *K*_M_, and the specificity constant *k*_cat_/*V*_max_ were determined by using gnuplot^1^. The specificity constant was directly assessed by applying the method of Johnson (2019). Supplementary Figures 9–18 give an overview of the obtained rate parameters described in Table 1. All raw data of the velocities and their average values are available in the Supplementary Data, Section “Kinetic Raw Data.”

**TABLE 1:** Kinetic parameters.

Substrate^1^	*K*_M_^2^ (nM)	*V*_max_ (nM/min)	*k*_cat_ (min^–1^)	*k*_cat_/*K*_M_^3^ (min^–1^ nM^–1^) × 10^–6^
C⋅C	220 ± 40	0.43 ± 0.03	0.0009 ± 0.0001	4.0 ± 0.4
C⋅C	1100 ± 300	2.0 ± 0.4	0.0040 ± 0.0008	4.0 ± 0.2
T⋅T	400 ± 200	0.9 ± 0.2	0.0018 ± 0.0004	5 ± 1
m*^*N*^*^4^C⋅C	190 ± 30	0.55 ± 0.04	0.0011 ± 0.0001	6.0 ± 0.6
m^5^C⋅C	500 ± 100	2.0 ± 0.3	0.0040 ± 0.0006	8.0 ± 0.8
m^5^C⋅T	1200 ± 300	1.3 ± 0.3	0.0026 ± 0.0006	2.0 ± 0.2
m*^*N*^*^4,5^C⋅C	1900 ± 300	8 ± 1	0.015 ± 0.002	8.0 ± 0.2
dHT⋅A	700 ± 300	3 ± 1	0.006 ± 0.002	10 ± 2
Tg⋅A	500 ± 300	1.8 ± 0.7	0.0036 ± 0.0001	7 ± 2
oxo^8^G⋅C	800 ± 200	5.0 ± 0.8	0.010 ± 0.002	128 ± 1

*^1^The lesion-containing or labeled strand is the first in each pair (usually in red; only for C⋅C also in blue, second pair. See Figure 1A). ^2^Determined using v_0_ = V_max_[S]/(K_M_ + [S]) with V_max_ and K_M_ fitted to all raw data. See Supplementary Figures 9–18 and Supplementary Data, Section “Kinetic Raw Data.” ^3^Determined by the method described in Johnson (2019). See Supplementary Figure 9.*

## Results

### Fpg Incises (Methyl)C⋅C, Methyl-C⋅T, and T⋅T Mismatches in DNA

5′-Fluorescently labeled polydeoxyribonucleotide substrates, with C, m^5^C, or m*^*N*^*^4^C inserted at a specific position, were prepared to study the potential enzymatic removal of these bases from DNA. Each ssDNA oligomer was annealed to a complementary strand, the above defined residue being placed opposite non-cognate C or T (unmethylated C placed opposite all four DNA bases; Figure 1A). They were then treated with *E. coli* Fpg. Base excision with the mono-functional Ung results in a NaOH-labile AP site. The bi-functional Fpg glycosylase, however, cleaves the AP site after base release, its activity monitored therefore on gels and without NaOH/heat treatment (Supplementary Figure 1). We, surprisingly, observed that Fpg exhibited significant activity for (unmethylated) C opposite C (Figure 1B, lane 6) and very low activity for C opposite T (lane 8; see Figure 1I). Virtually no activity was, however, detected opposite A (lane 5) and G (lane 7). Incubation of the substrates alone and without enzyme as a negative control, followed by NaOH/heat treatment, resulted in no product band (Figure 1B, lanes 1–4). This confirmed the dependence of the observed activity on Fpg, and demonstrated the integrity of the substrate. Additional control incubations with U⋅G-DNA alone (Figure 1B, lane 9) and with Ung (lane 10) and Ung plus Fpg, demonstrated active Fpg (i.e., AP lyase activity; lane 11). The experiments with methylated C opposite C showed the highest Fpg activity for m*^*N*^*^4,5^C (Figure 1C, lane 6), and lower and similar activities for m*^*N*^*^4^C (lane 4) and m^5^C (lane 5). The negative control experiments gave no product band, as previously (Figure 1C, lanes 1–3). Fpg furthermore showed similar activity levels for m^5^C and m*^*N*^*^4,5^C if placed opposite T in DNA (Figure 1D, lanes 3 and 4), activity being clearly lower than opposite C, particularly for m*^*N*^*^4,5^C (Figure 1C, lanes 5 and 6, respectively). The negative control experiments also gave no product (Figure 1D, lanes 1 and 2). We, at this point of the investigation, asked ourselves whether base sequence is a determinant of Fpg activity, which so far was determined using lesion X in the context of 5′-ACXAG-3′. This encouraged us to measure the enzyme activity of C or m^5^C (lesion Y) opposite C, by labeling the other strand of the substrate, the first labeled strand being kept unlabeled (Figure 1A). This resulted in the 5′-CTYGT-3′ context. As before, the results showed higher Fpg activity for m^5^C opposite C (Figure 1E, lane 4) than for C opposite C (lane 3). The substrate not treated with Fpg showed no cleavage (lanes 1 and 2) and the U⋅G control substrate with Ung demonstrated active Fpg (lanes 5–7). The results also showed an insignificant difference between the Fpg activity of m^5^C and C opposite C in the two sequence contexts (Figure 1H). We, to conclude the investigation and compare our results on C and methylated Cs with known Fpg substrates, analyzed oxo^8^G, Tg, and dHT in the same sequence context. T⋅T, A⋅A, and G⋅G were also included as substrates to examine whether C⋅C is the only homo-mismatch targeted by Fpg (Figure 1A). The results confirm high Fpg activity for oxo^8^G, almost all substrate being cleaved (Figure 1F, lane 12). Significant (although much lower) background cleavage was, however, observed following NaOH/heat treatment without Fpg (lane 6). This indicates the spontaneous release of oxo^8^G from DNA during storage and/or preparation of the substrate. Fpg also caused nearly complete cleavage at the Tg (Figure 1G, lane 5) and dHT (lane 6) sites in DNA. Cleavage at the C⋅C mismatch (lane 4) was, in comparison, much less evident. The background cleavage at Tg (lane 2) and dHT (lane 3) was less than at oxo^8^G (Figure 1F, lane 6), no such cleavage occurring at the C⋅C site (Figure 1G, lane 1). A robust AP lyase function confirmed, as previously, an active enzyme (Figures 1F,G, lanes 13–15 and 7–9, respectively). Surprisingly, Fpg incised at the T⋅T mismatch with a similar efficiency (Figure 1F, lane 10) as at C⋅C (lane 8). This contrasts with the very low activity for the A⋅A and G⋅G homo-mismatches (Figures 1F,J lanes 7 and 9). No background cleavage was observed in these cases (lanes 2, 4, 1, and 3, respectively). In conclusion, the substrates showed the following susceptibility to being cleaved by Fpg: oxo^8^G⋅C > Tg⋅A, dHT⋅A > m*^*N*^*^4,5^C⋅C > m^5^C⋅C, m*^*N*^*^4^C⋅C > m*^*N*^*^4,5^C⋅T, m^5^C⋅T > T⋅T, C⋅C > G⋅G, A⋅A, C⋅T > C⋅G, C⋅A (Figures 1H–J). Activity therefore increases when the targeted cytosine base is enzymatically methylated in the order m*^*N*^*^4,5^C > m^5^C, m*^*N*^*^4^C > C.

### Mismatch Incision Activity of Fpg Confirmed by Imine Enzyme–DNA-Deoxyribose Intermediate Formation

**FIGURE 2:**
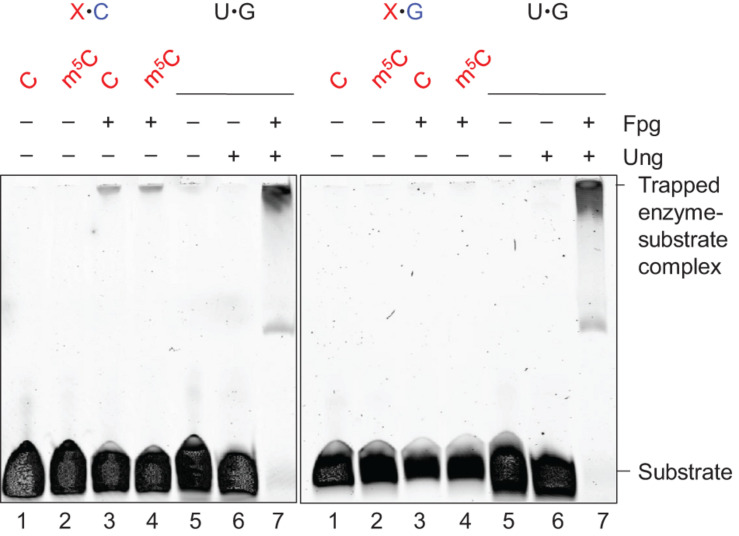
Schiff base trapping analysis of Fpg protein. DNA substrate (1 pmol) with either X⋅C (left panel) or X⋅G (right panel) base pair, alone as a negative control (lanes 1 and 2), or together with Fpg (10 pmol; lanes 3 and 4), was incubated with 50 mM NaBH_4_ in reaction buffer at 37°C for 1 h (final volume, 10 μL). U⋅G-DNA (30 nt; 1 pmol) incubated alone (lane 5), with Ung (10 pmol; lane 6), or with Ung and Fpg (10 pmol each; lane 7), was used as a negative and positive control for active Ung and Fpg, respectively, Ung converting U⋅G-DNA into AP-DNA to be trapped by Fpg. The trapped protein was separated from un-trapped protein by denaturing PAGE. The experiments were performed 10 (X⋅C) or 5 times (X⋅G), the result being the same.

Formamidopyrimidine-DNA glycosylase is a bi-functional DNA glycosylase. It therefore forms an imine enzyme–DNA-deoxyribose (Schiff base) intermediate with the DNA substrate (Zharkov et al., 1997), and can be stably cross-linked to it after being treated with sodium borohydride (which reduces the double bond of the complex). We performed these experiments with an enzyme concentration 10 times higher than the substrate concentration at 1 h incubation time. The results showed that Fpg forms such a complex with the C⋅C (Figure 2, left panel, lane 3) and m^5^C⋅C substrates (lane 4) and with the AP-DNA used as a positive control (left and right panels, lane 7). This confirms that active Fpg exhibits activity for C and m^5^C opposite C. No trapped complex was observed following incubation of the C⋅C and m^5^C⋅C substrates without enzyme (Figure 2, left panel, lanes 1 and 2, respectively), following incubation with the U⋅G substrate alone (lane 5) or with only Ung (lane 6). No trapped complex was formed following incubation of the C⋅G and m^5^C⋅G substrates, with (Figure 2, right panel, lanes 3 and 4) or without Fpg (lanes 1 and 2). This confirms the specific nature of Fpg activity for the (methyl)C⋅C mismatch.

### Mismatched DNA Incised by Fpg Is Processed by Downstream BER Proteins

**FIGURE 3:**
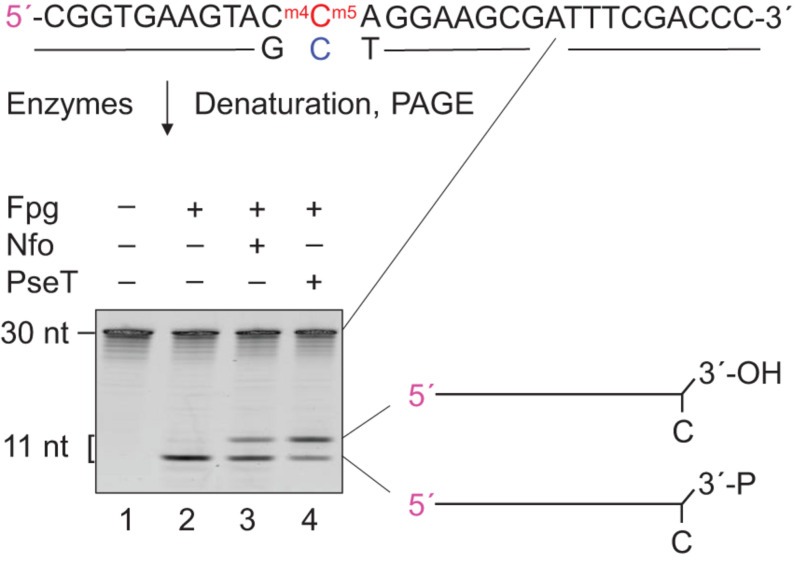
Definition and processing of the 3′-end after Fpg-mediated incision of m*^*N*^*^4,5^C⋅C-DNA. DNA substrate (Figure 1A; 1 pmol) was incubated without (lane 1) or with Fpg (13 pmol; lanes 2–4) at 37°C for 30 min, followed by no addition (lanes 1 and 2), addition of 0.083 pmol endonuclease IV (Nfo; lane 3) or addition of 0.29 pmol T4 polynucleotide kinase (PseT; lane 4), and incubation for an additional 30 min (final volume, 10 μL). Incised DNA was separated from un-incised DNA by denaturing PAGE (Supplementary Figure 1) at 500 V for 4 h. Abbreviation: 3′-P, 3′-phosphate. The 5′-labeled strand is indicated by 5′ in magenta.

Apurinic/apyrimidinic lyase activity of Fpg leaves a 3′-phosphate (Bailly et al., 1989), which is further processed by BER proteins (Friedberg et al., 2006). We incubated Fpg-treated m*^*N*^*^4,5^C⋅C-DNA (Figure 1A) with T4 PseT, to verify the presence of a 3′-phosphate in (methyl)C⋅C-DNA following the incision by Fpg. This specifically removes phosphate from the 3′-end (Cameron and Uhlenbeck, 1977; Midgley and Murray, 1985). As a result, a slower-migrating product corresponding to a 3′-OH product was generated (Figure 3, lane 4) in PAGE under conditions that favor the separation of different end-products. Incubation without enzyme formed no product (Figure 3, lane 1) and incubation with Fpg alone only formed 3′-phosphate (lane 2). This confirms that the Fpg-mediated incision of m*^*N*^*^4,5^C⋅C-DNA forms a 3′-phosphate. Incubation with Fpg followed by the addition of endonuclease IV (Nfo) (Warner et al., 1980; Doetsch and Cunningham, 1990) also formed 3′-OH product (Figure 3, lane 3), showing that the (methyl)C⋅C-DNA incised by Fpg is processed by downstream BER proteins, which is in agreement with previous knowledge (Friedberg et al., 2006). This supports the role of Fpg as an *E. coli* DNA glycosylase that initiates the repair of C⋅C-mismatched DNA (Supplementary Figure 2; Doetsch and Cunningham, 1990; Patel et al., 2001; Chauleau and Shuman, 2016).

### Efficient Target Binding Is Followed by Slow Incision

A further aim was to conduct a thorough kinetic analysis of the activity of Fpg for the different forms of C opposite C, and T opposite T. A suitable Fpg concentration for performing multiple turnover kinetic analysis had, however, to be first determined. m^5^C⋅C-DNA was chosen as an “average targeted substrate” for this (Figure 1H), 50 nM being exposed to different concentrations of the enzyme for an increasing period of time of up to 1 h. We, based on the results (Supplementary Figure 3A), chose 500 nM as a suitable Fpg concentration, the incubation time being 30 min. Kinetic analysis was furthermore performed on six of the most efficient new Fpg substrates (except m*^*N*^*^4,5^C⋅T), including both Cs of the C⋅C mismatch and the previously described substrates oxo^8^G, Tg, and dHT (Figures 1H–J). The results showed incision activity that in all cases corresponds to a graph that describes *Michaelis–Menten behavior* (Supplementary Figures 3B–K). The kinetic parameters largely confirm the initial experiments cited above, and show that m^5^C⋅C and m*^*N*^*^4,5^C⋅C exhibit the highest *k*_cat_/*K*_M_ followed by m*^*N*^*^4^C⋅C and then C⋅C. All values are, however, mostly within the same order of magnitude. Both Cs of the C⋅C mismatch seemed to be almost identically processed by Fpg (Figure 1H), as confirmed by their identical *k*_cat_/*K*_M_ value (Table 1). Their *K*_M_ and *V*_max_ values, however, varied by a factor of 5, this showing the ease or strength by which Fpg recognizes a C⋅C, and that the rate of incision is dependent on the sequence context. The higher *K*_M_ value in one context is compensated by a higher *k*_cat_ in the other, this resulting in an identical *k*_cat_/*K*_M_ value. It should also be noted that only one of the bases in the (methyl)C⋅C mismatches investigated is present on the labeled strand. This renders the activity of the opposite base undetectable (Figure 1A). Most kinetic constants measured therefore underestimate the total Fpg activity of a specific mismatch. This contrasts with the C⋅C mismatch, where each strand is labeled and monitored in separate experiments (Supplementary Figures 3B,G), resulting in a *V*_max_ and *k*_cat_ for both Cs (Table 1). The highest Fpg activity was, as expected, exhibited for oxo^8^G with a *k*_cat_/*K*_M_ value one order of magnitude higher than for all other substrates (Table 1). It is, however, interesting to note that this difference is mainly due to the *k*_cat_ values, the *K*_M_ values being similar for all substrates. This indicates that Fpg recognizes the C⋅C and T⋅T mismatches as efficiently as it recognizes oxo^8^G in DNA. The *K*_M_ values determined for C⋅C and T⋅T are similar to the values previously determined for dHT (450 nM). They are also one order of magnitude lower than the *K*_M_ values for 5-hydroxycytosine (4,700 nM) while inserted into a similarly sized (33 nt) DNA oligomer (D’Ham et al., 1999). These kinetic parameters are quite close to other previously determined *K*_M_ and *k*_cat_ values for Fpg (Karakaya et al., 1997; Sidorkina et al., 2001).

### Fpg Targets Only One of the Cs in a C⋅C Mismatch

The targeting of the C⋅C mismatch by Fpg is most probably indiscriminate. It could therefore be theoretically possible that the excision/incision event of the first C/AP site is followed by an excision/incision of the second C/AP site, resulting in a ds-break. A double length C⋅C-DNA (60 nt; Supplementary Figure 4), which has a greater resistance to denaturation, was therefore treated with Fpg using the same controls and experimental conditions as before (see Figure 1). The Fpg-incised DNA was, however, subjected to both non-denaturing and denaturing PAGE and monitored for possible ds breakage. Both should show a 39 nt length cleavage product if this is the case (Supplementary Figure 4, left panel). However, no such incision product was observed following a non-denaturing PAGE, not in the control incubations without the enzyme (Supplementary Figure 4, green square, lanes 1 and 2) nor with Fpg (lanes 3 and 4). This indicates that Fpg is unable to form a ds-break during the processing of the C⋅C mismatch in DNA, the results from denaturing PAGE showing that Fpg only incises one of the DNA strands (Supplementary Figure 4, brown square, lanes 7 and 8). This apparent inability of Fpg to form a ds-break in DNA by simultaneously targeting both Cs of the C⋅C mismatch, is supported by present knowledge. Fpg exhibits no activity for its major substrate oxo^8^G in ssDNA (Tchou et al., 1991) which, together with its preference for C as the opposite base (Fromme and Verdine, 2002), confirms the dsDNA requirement. A possible rotation to accommodate this second C without this anchor does not, however, accord with the “searching model” for Fpg finding the substrate base and forming the “lesion recognition complex” (Boiteux et al., 2017). This further supports our result.

### Rate Parameters

The determined kinetic parameters are shown in Table 1. The Supplementary Figures 9–18 give an overview of the results.

## Discussion

### Fpg Is a Pyrimidine⋅Pyrimidine Mismatch DNA Glycosylase

Most mismatches arising in DNA by replication errors, are repaired by the extensively described MMR system. This system consists, across species, of orthologous proteins. The C⋅C mismatch is, however, an exception (Friedberg et al., 2006; Iyer et al., 2006). The MMR system may, in some model organisms such as *Saccharomyces cerevisiae* and *Schizosaccharomyces pombe*, be proficient in repairing C⋅C (Bowen et al., 2013; Srivatsan et al., 2014). This is, however, believed to be a very poor substrate for MMR in *E. coli* and mammalian cells (Su et al., 1988), spurring the search for other repair strategies in these organisms (Nakahara et al., 2000; Muheim-Lenz et al., 2004). A previous report that shows the binding of Fpg to the C⋅C mismatch in DNA (Nakahara et al., 2000), and our recent discovery that the enzyme exhibits activity for a doubly methylated cytosine opposite C and T (Alexeeva et al., 2018), urged us to systematically investigate Fpg’s capacity to incise the different methylated forms, including unmethylated C opposite the two pyrimidines in DNA. We, interestingly, found Fpg activity for C⋅C, methyl-C⋅C, methyl-C⋅T, and also for the T⋅T mismatch. Incision activity was, however, one order of magnitude lower than for oxo^8^G, the primary oxidized base removed by the enzyme (Table 1). Our findings, nevertheless, significantly broaden the known substrate specificity of this enzyme.

### Fpg Activity for (methyl)C⋅C Is Indicated by Structural Considerations

Formamidopyrimidine-DNA glycosylase exhibits activity for an array of oxidized bases (Bjelland and Seeberg, 2003) and for unmodified pyrimidines and methylated cytosines. Explaining this promiscuous nature of Fpg, however, requires an unrestricted active site. Current knowledge demonstrates that Fpg has an open and flexible active site pocket (Fromme and Verdine, 2002, 2003; Serre et al., 2002; Amara et al., 2004; Coste et al., 2004, 2008; Perlow-Poehnelt et al., 2004), the base recognition cavity having a flexible “lid,” the so-called αF–β9 loop (Fromme and Verdine, 2003; Coste et al., 2004), which is structurally ordered in some solved structures and disordered in others. The “lid” closes over different substrate lesions in different ways, and is therefore able to recognize very different bases. The loop’s amino acid sequence is, even so, not conserved in the organisms investigated. This is probably because the lesions primarily bind to the loop, and via H-bonds to backbone amides, rather than to side chains (Fromme and Verdine, 2002; Serre et al., 2002; Zharkov et al., 2003). This is shown in the crystal structures of oxo^8^G (PDB code: 1R2Y) and dHU (PDB code: 1R2Z) with the *Geobacillus stearothermophilus* Fpg. The *O*^6^ of guanine in oxo^8^G⋅C forms four H-bonds to the backbone amides of Val222, Arg223, Thr224, and Tyr225 (numbering corresponds to the *G. stearothermophilus* Fpg) (Fromme and Verdine, 2003). N1 is, furthermore, a H donor to the side chain of Thr224, *N*^2^ is a H donor for Thr224 and Glu78, and N7 is a H donor for Ser220. *O*^4^ and N3 of dHU occupy positions in the crystal structure that are similar to *O*^6^ and N7 of oxo^8^G, respectively. We suggest that the targeted cytosine in the C⋅C mismatch forms H-bonds with the key lesion binding residues at the beginning of the αF–β9 loop, *N*^4^ being a donor and N3 and *O*^2^ being acceptors, as demonstrated by the crystal structures of the similarly sized lesions mentioned above (PDB codes: 1R2Z and 1R2Y) (Supplementary Figure 5; Fromme and Verdine, 2002). The *K*_M_ values of Fpg for C⋅C at the forward (Table 1, first line) and reverse (Table 1, second line) strands, demonstrate a significant difference in affinity, and indicate the importance of the sequence context in the recognition of substrate. *N*^4^-methylation of C causes a slightly lower *K*_M_, probably due to interference with the hydrophobic side chain of Leu216, Thr215, or Thr214 in the αF–β9 active site loop (Supplementary Figure 5). 5-Methylation, however, causes a 50% decrease and double methylation causes a decrease of one order of magnitude in substrate binding, as indicated by this parameter.

The steps of the catalytic mechanism for Fpg are defined. They consist of: (1) formation of an “encounter complex” where Fpg binds to the intrahelical lesion in DNA, (2) formation of the “lesion recognition complex” where the lesion flips out and binds to the active site loop, (3) excision of the extrahelical lesion by nucleophilic attack on deoxyribose C′1 by the N-terminal Pro1, and (4) incision of the DNA strand by conjugate elimination leading to the formation of 3′- and 5′-phosphate ends and the removal of the sugar moiety. The same residues (i.e., Pro1 and Glu2) are involved in the excision and incision step of different lesions. Light has been shed on the molecular details of “encounter” and “lesion recognition complex” formation by the crystal structures of Fpg orthologs from different microorganisms in complex with oxo^8^G, dHU, or 2,6-diamino-4-hydroxy-5-formamidopyrimidine. The enzyme kinetics of Fpg show a strong preference for the damaged base opposite C, oxo^8^G being opposite C rather than opposite A. Fpg introduces strong torsion by inserting Met73, Phe110, and Arg108 in the intrahelical space of the oxo^8^G⋅C pair, so ensuring the extrusion of oxo^8^G and preventing its reinsertion into the DNA groove. A similar mechanism most likely explains the extrusion of the encountered C in the C⋅C mismatch. Phe110 therefore buckles the lesion, the thioether side chain of Met73 acting as a steric block that stabilizes the extrahelical conformation via van der Waals interaction with the sugar moiety and the phosphate backbone flanking the lesion. Arg108 stabilizes the opposite C by providing two H-bonds, respectively, from Nη and Nε to N3 and *O*^2^ of cytosine. The opposite C is further stabilized by Arg109 contact with the DNA backbone. The C⋅C mismatch, significantly, destabilizes the DNA helix thermodynamically in the range of 7–9 base pairs around the lesion (Tikhomirova et al., 2006). We speculate that this can be detected by Fpg during its interrogation of DNA, via residue Phe110. In summary, stabilization of the opposite C by Arg108 and Arg109, occupation of the intrahelical space by Phe110 and interaction of Met73 with the backbone of the lesion, allow the extrusion of the damaged base into the active site loop αF–β9. We determined that the *k*_cat_ of Fpg for m*^*N*^*^4,5^C⋅C is higher than for C⋅C (Table 1). This accords with the strong helix-disrupting capability of m*^*N*^*^4,5^C that we discuss elsewhere (Alexeeva et al., 2018). This can be further explained by the introduction of methyl groups and by this leading to stronger hydrophobic disturbance in the intrahelical space. This therefore facilitates the buckling and extrusion of m*^*N*^*^4,5^C by Phe110 and Met73, this not taking place in unmodified C. Like T opposite oxo^8^G (Bjelland and Seeberg, 2003; Zharkov et al., 2003), Fpg accepts T opposite the (methylated) Cs (Figure 1I). It therefore seems some flexibility in the conformation of the estranged base is retained at the Fpg active site, also with these substrates.

### Fpg May Function in the Removal of the Destabilizing C⋅C Mismatch From Cellular DNA

We, based on previous knowledge of replicative and TLS Pols in *E. coli* and the generation of the C⋅C mismatch (Wagner and Nohmi, 2000; Kuban et al., 2005; Fijalkowska et al., 2012; Fuchs and Fujii, 2013), suggest the following working model to summarize the expected origin of a C⋅C mismatch in *E. coli* DNA and its putative destiny (Supplementary Figure 6). Pol III normally replicates the genome by high level of processivity and fidelity (step 1), so removing base mismatches efficiently (steps 2 and 3) (Fijalkowska et al., 2012). A C⋅C mismatch that evades these defenses might, despite this being very unlikely, be extended by Pol III (step 4). Pol III then leaves the β clamp used by Pol IV (step 5) to continue synthesis. Such Pol switching or replacement of Pol III by a TLS Pol is thought to occur more often during lagging than leading strand synthesis. The TLS path should have a length (≥5 nt) which is sufficient to avoid 3′→ 5′ exo degradation by the re-recruited Pol III. Genomic stress conditions that induce the SOS response should favor C⋅C persistence, by increasing the dNTP pool size which promotes Pol III synthesis rather than exo activity, and by increasing the level of Pol IV. Previous results and the results presented here indicate two possible scenarios following Pol IV departure. Either the C⋅C mismatch survives the ongoing round of replication and Pol III replicates both strands, resulting in a G⋅C → C⋅G mutation in 50% of the offspring (step 6a and b). Or Fpg is recruited to C⋅C, so initiating BER (step 7). The opposed Cs are then selected randomly, unless Fpg is not somehow guided by other proteins to recognize the incorrect C, the cost being a point mutation of 50% of repair events (step 7a and b). We hypothesize that this mismatch-DNA glycosylase activity of Fpg may eliminate an unprotected and nuclease-sensitive structure that is destined to threaten DNA integrity, and therefore represents a survival strategy that is as acceptable as other error-prone processes such as TLS (Supplementary Figure 6).

As for all types of base substitutions, the G⋅C → C⋅G transversion can be formed by the insertion of a G opposite G or a C opposite C during DNA replication. This is due to Pol error, damage to the template or because the inserted base alters the pairing abilities. G⋅C → C⋅G is the rarest base substitution formed in wild-type *E. coli* (Foster et al., 2015). This might be partly due to the MutHLS system being efficient toward the G⋅G mismatch. However, the same rarity is observed in Pol III exo^–^ cells (Niccum et al., 2018), this suggesting that the ability of Pol III to insert C opposite C and to insert G opposite G is limited. This may partly explain why MMR did not evolve to repair the C⋅C mismatch (Iyer et al., 2006). C⋅C, when it occasionally forms during replication, may instead be extended by Pol IV and eventually resolve into a stable G⋅C pair in the next replicative event. If the cells move into more stationary conditions, then a persisting and destabilizing C⋅C mismatch is targeted by Fpg and resolved by BER. In both scenarios (i.e., Fpg present or not) the cost is 50% mutated offspring. This accords with the similarly low levels of G⋅C → C⋅G transversions demonstrated in wild-type and *fpg*^–^ cells (Cabrera et al., 1988; Kuipers et al., 1999).

### The Biological Impact of Fpg Activity Upon Methylated Cs Is Elusive

m^5^C is, in protecting against DNA cleavage in restriction–modification systems and in DNA repair, as important a canonical methyl-base modification in prokaryotic DNA as m*^*N*^*^4^C and *N*^6^-methyladenine. All three base modifications are products of the enzyme-catalyzed transfer of a methyl group from *S*-adenosyl-L-methionine to the cognate unmodified base by a DNA methyltransferase (MTase) (Jeltsch and Jurkowska, 2016). Most prokaryotes contain either m^5^C or m*^*N*^*^4^C in their DNA. Some contain both (Janulaitis et al., 1983; Ehrlich et al., 1987). Insertion of a C opposite template m^5^C or m*^*N*^*^4^C is an additional source of C⋅C mismatch analogs. m^5^C has been shown to be a substrate for enzymatic *N*^4^-methylation (Butkus et al., 1985; Klimašauskas et al., 2002). m*^*N*^*^4,5^C may therefore also occur in certain cells containing MTases of both positions. The biological relevance of Fpg in resolving mismatches that involve m^5^C, m*^*N*^*^4^C and in particular m*^*N*^*^4,5^C, and that are expected to have very much lower *in vivo* abundance than C⋅C and T⋅T, should be limited. Their possible presence and impact are therefore largely unknown and might represent a call for further assessment. Another perspective is that Fpg was selected during evolution to remove a variety of DNA base lesions, including the very destabilizing C⋅C mismatch, irrespective of whether they are methylated or not.

## Conclusion

We here, for the first time, report that the *E. coli* Fpg protein exhibits *in vitro* DNA glycosylase activity for C opposite C (Figure 1). This initiates BER of this most destabilizing and MMR resistant mismatch in DNA, putatively at the partial cost of G⋅C → C⋅G transversion (Supplementary Figure 6). Similar activity was demonstrated for T⋅T, which is also a substrate for the *E. coli* MMR system (Iyer et al., 2006). This represents an argument against Fpg being a significant alternative to its immediate post-replicative resolution. Fpg may, however, play a role in resolving both C⋅C and T⋅T when Dam has methylated the GATC sites on both strands following DNA replication. The corresponding *K*_M_ values indicate efficient targeting by Fpg, despite low turnover numbers for the incision of C⋅C and T⋅T (Table 1). It has been reported that the nucleoid-associated HU protein facilitates enzyme release (Le Meur et al., 2015) if low *k*_cat_ is partially due to the strong binding of Fpg to the incised site in DNA (Boiteux et al., 2017). Further studies should therefore include the investigation of the possible enhancement of Fpg activity and damaged strand recognition, by collaboration with other factors including (but not limited to) HU and downstream BER proteins.

## Data Availability Statement

The original contributions presented in the study are included in the article/Supplementary Material, further inquiries can be directed to the corresponding author/s.

## Author Contributions

AT designed and performed most of the experiments, analyzed most of the data, supervised parts of the experiments, and wrote parts of the manuscript. MA designed and supervised parts of the experiments, analyzed the structural implications of the data, and wrote parts of the manuscript. MT produced experimental tools and contributed to important intellectual content. AA and PG designed and performed parts of the experiments and analyzed the data. AK initiated the study and contributed to important intellectual content. SK initiated the study, suggested important experiments, and contributed to important intellectual content. PR performed the kinetic analysis and wrote the manuscript. SB initiated the study, designed the experiments, analyzed the data, supervised and managed the study, and wrote the manuscript. All the authors read, commented on, and revised the manuscript.

## Conflict of Interest

The authors declare that the research was conducted in the absence of any commercial or financial relationships that could be construed as a potential conflict of interest.

## Footnotes

1 http://www.gnuplot.info
