# Supplementary material for: Alleviation of C⋅C Mismatches in DNA by the Escherichia coli Fpg Protein
Source: Front Microbiol. 2021 Jun 30;12:608839. doi: 10.3389/fmicb.2021.608839 (PMC8278400; doi:10.3389/fmicb.2021.608839)
Supplement: Supplementary file 1 [file Data_Sheet_1.docx]

Supplementary Material

# Supplementary Data

**Validity of the Michaelis-Menten Approach**

***Irreversible Michaelis-Menten Equation***

**The rate equations of the irreversible Michaelis-Menten mechanism**

***k*_1_ *k*_2_**

**E + S ⇄ E**S → E + P (1)

***k***_–_**_1_**

were analyzed numerically using the FORTRAN subroutine LSODE (Radhakrishnan and Hindmarsh, 1993):

*Ė* = –*k*_1_ [E] [S] + (*k*_–1_ + *k*_2_) [ES]

$\frac{d[ES]}{dt}$ = –*Ė*

*Ṡ* = –*k*_1_ [E] [S] + *k*_–1_ [ES]

*Ṗ* = *k*_2_ [ES]

where$\dot{X}$= $\frac{dX}{dt}$ is the time derivative of compound X concentration.

The numerical solutions were compared with approximations, based on the rapid equilibrium between E, S, and the enzyme-substrate complex ES, and on the steady-state approximation that time derivatives of [E] and [ES] are zero (Segel, 1975).

The numerical, steady-state (ss), and rapid equilibrium (re) expressions for the reaction velocity of Equation (1) were calculated as:

*v*_num_ = *k*_2_ [ES]

*v*_ss_ = $\frac{V\text{max } \left[ S \right]}{K\text{M}\text{ss} + \left[ S \right]}$

*v*_re_ = $\frac{V\text{max } \left[ S \right]}{K\text{M}\text{re} + \left[ S \right]}$

where *k*_2_ is the turnover number, *V*_max_ = *k*_2_ ∙ [E]_tot_, and [E]_tot_ is total enzyme concentration. The *K*_M_ values for the steady-state and rapid equilibrium approximations are, according to (Segel, 1975), given as:

*K*_M_^ss^ = $\frac{\left[ E \right] \left[ S \right]}{\left[ \mathrm{ES} \right]}$ = $\frac{k\text{–1 }+ k\text{2}}{k\text{1}}$

*K*_M_^re^ = $\frac{\left[ E \right] \left[ S \right]}{\left[ \mathrm{ES} \right]}$ = $\frac{k\text{–1 }}{k\text{1}}$

***The Steady-State Approximation Implies Pseudo-First-Order Kinetics with Respect to S***

**The steady state approximation,** **d[ES]/dt = 0, implies that the reaction velocity v = *k*_2_[ES] is first-order with respect to S. In other words, [S] decreases exponentially with time. This can be seen by the time derivative of the following mass balance**

**[S]_0_ = [P](t) + [S](t) +** **[ES](t)**

**where [S]_0_ is the initial concentration of substrate S at time t = 0. We assume, for the sake of simplicity, that no product is present at time t = 0. [S](t), [P](t) and [ES](t) are the concentrations of S, P and ES at time t. The time derivative of the above mass balance and the assumption that d[ES]/dt = 0 gives**

*Ṗ* + *Ṡ* = 0

**which implies that** *v*_pfo_ = *Ṗ* = –*Ṡ* = *k*_2_ [ES] = $\frac{k\text{2 }\left[ E \right]}{K\text{M}}$ ∙ [S] = *k*_pfo_ ∙ [S]

Noting that [ES] = [E][S]/*K*_M_, that the steady-state approximation leads to constant [E], and that the pseudo-first-order (pfo) rate constant *k*_pfo_ is therefore constant, we can write

*Ṡ* = *k*_2_ [ES] = –*k*_pfo_ ∙ [S] ⇒ [S](t) = [S]_0_ ∙ $e^{-k}\text{pfo}\text{}\text{t}$

i.e. [S](t) shows an exponential decrease with time.

***Range of Rate Constants Considered***

As previously described (Segel, 1975), the values of *k*_1_, *k*_–1_, and *k*_2_ generally lie within the following ranges:

*k*_1_, 10^7^–10^10^ M^–1^ min^–1^

*k*_–1_, 10^2^–10^6^ min^–1^

*k*_2_, 50–10^7^ min^–1^

We in **Supplementary Table 2** use combinations of the upper and lower values of these to test the steady-state and rapid equilibrium approximations against the numerical calculation of substrate and total enzyme concentrations.

***Validity of the Michaelis-Menten Approach***

It can be questioned whether the Michaelis-Menten approach indicated above is still valid when [E]_tot_ = 500 nM and [S]_0_ is as low as 5 nM. We, in the following, show that it is still valid and that the equation

*v*_0_ = $\frac{V\text{max } \left[ S \right]}{K\text{M} + \left[ S \right]}$

gives an excellent description of velocities compared with numerically calculated velocities *v*_num_. We have tested eight rate constant combinations (see **Supplementary Table 2**) and found that the steady state approximation of seven of these gives excellent agreement with *v*_num_. Only the rapid equilibrium approximation in combination No. 6 gives slightly better agreement than the steady-state approximation. *v*_re_ is, also for No. 6, in excellent agreement with *v*_num_. This is shown in **Supplementary Figures 7 and 8** in which the numerical solutions of the rate constant combinations from **Supplementary Table 2** are compared with the results of the steady state and rapid equilibrium approximations (upper panels). The lower panels show the corresponding velocities.

***Experimental Velocity Data Agrees with First-Order Kinetics***

The experimentally determined velocity data (**Supplementary Data**, section “Kinetic Raw Data”) agrees well with the above implicated first-order kinetics**. First-order kinetics implicate that the same fraction of substrate is processed irrespective of the initial concentration of the substrate, during the 30 min assay period, which was observed** (**Supplementary Table 3)**. The rate constant *k*_pfo_ can be derived from the following relationship

*k*_pfo_ = – $\frac{1}{t\text{assay}}$ ∙ ln ($\frac{[S]}{[S]\text{0}}$)

where *t*_assay_ is the assay time (30 min) and [S]/[S]_0_ is the ratio of remaining S at the end of the assay time.

**Kinetic Raw Data**

C**∙**C

| **[S]** | |  |  | **velocity, nM/min** | |  |
| --- | --- | --- | --- | --- | --- | --- |
| **pmol/20 µL** | | **nmol/20 µL** | **nM** | **Average** | **SD** |  |
| 0.125 | | 0.000125 | 6.3 | 0.0031192 | 0.003 |  |
| 0.25 | | 0.00025 | 13 | 0.0074616 | 0.001 |  |
| 0.5 | | 0.0005 | 25 | 0.0291911 | 0.005 |  |
| 1 | | 0.001 | 50 | 0.0896232 | 0.031 |  |
| 2 | | 0.002 | 100 | 0.1262553 | 0.032 |  |
| 4 | | 0.004 | 200 | 0.2175025 | 0.029 |  |
| 8 | | 0.008 | 400 | 0.2617096 | 0.025 |  |
| 10 | | 0.01 | 500 | 0.3001807 | 0.024 |  |
| **Parallel 1** |  |  |  |  | |  |
| [S] |  |  |  |  | |  |
| pmol/20 µL | nmol/20 µL | nM | cleavage, % | cleavage, nM | | velocity, nM/min |
| 0.125 | 0.000125 | 6.25 | 0.31509612 | 0.019693508 | | 0.00065645 |
| 0.25 | 0.00025 | 12.5 | 1.652535175 | 0.206566897 | | 0.006885563 |
| 0.5 | 0.0005 | 25 | 2.738026842 | 0.684506711 | | 0.02281689 |
| 1 | 0.001 | 50 | 3.601123084 | 1.800561542 | | 0.060018718 |
| 2 | 0.002 | 100 | 2.679120363 | 2.679120363 | | 0.089304012 |
| 4 | 0.004 | 200 | 3.072264667 | 6.144529334 | | 0.204817644 |
| 8 | 0.008 | 400 | 1.831599231 | 7.326396925 | | 0.244213231 |
| 10 | 0.01 | 500 | 1.774749591 | 8.873747953 | | 0.295791598 |

C**∙**C

| **Parallel 2** |  |  |  |  |  |
| --- | --- | --- | --- | --- | --- |
| [S] |  |  |  |  |  |
| pmol/20 µL | nmol/20 µL | nM | cleavage, % | cleavage, nM | velocity, nM/min |
| 0.125 | 0.000125 | 6.25 | 2.42495357 | 0.151559598 | 0.005051987 |
| 0.25 | 0.00025 | 12.5 | 2.034719587 | 0.254339948 | 0.008477998 |
| 0.5 | 0.0005 | 25 | 4.071107411 | 1.017776853 | 0.033925895 |
| 1 | 0.001 | 50 | 5.175942793 | 2.587971397 | 0.086265713 |
| 4 | 0.004 | 200 | 3.894278132 | 7.788556264 | 0.259618542 |
| 10 | 0.01 | 500 | 1.673856683 | 8.369283415 | 0.278976114 |
| **Parallel 3** |  |  |  |  |  |
| [S] |  |  |  |  |  |
| pmol/20 µL | nmol/20 µL | nM | cleavage, % | cleavage, nM | velocity, nM/min |
| 0.125 | 0.000125 | 6.25 | 0.252880049 | 0.015805003 | 0.000526833 |
| 0.25 | 0.00025 | 12.5 | 1.890398588 | 0.236299824 | 0.007876661 |
| 0.5 | 0.0005 | 25 | 3.319284881 | 0.82982122 | 0.027660707 |
| 1 | 0.001 | 50 | 4.734319063 | 2.367159531 | 0.078905318 |
| 2 | 0.002 | 100 | 4.453775845 | 4.453775845 | 0.148459195 |
| 4 | 0.004 | 200 | 2.908557381 | 5.817114761 | 0.193903825 |
| 8 | 0.008 | 400 | 2.094044126 | 8.376176504 | 0.279205883 |
| 10 | 0.01 | 500 | 1.954645725 | 9.773228627 | 0.325774288 |
| **Parallel 4** |  |  |  |  |  |
| [S] |  |  |  |  |  |
| pmol/20 µL | nmol/20 µL | nM | cleavage, % | cleavage, nM | velocity, nM/min |
| 0.125 | 0.000125 | 6.25 | 2.995983805 | 0.187248988 | 0.006241633 |
| 0.25 | 0.00025 | 12.5 | 1.585507679 | 0.19818846 | 0.006606282 |
| 0.5 | 0.0005 | 25 | 3.883315523 | 0.970828881 | 0.032360963 |
| 1 | 0.001 | 50 | 7.998186124 | 3.999093062 | 0.133303102 |
| 2 | 0.002 | 100 | 4.230083569 | 4.230083569 | 0.141002786 |
| 4 | 0.004 | 200 | 3.175049849 | 6.350099698 | 0.21166999 |

C**∙**C

| **[S]** | |  |  | **velocity, nM/min** | | |  |
| --- | --- | --- | --- | --- | --- | --- | --- |
| **pmol/20 µL** | | **nmol/20 µL** | **nM** | **Average** | | **SD** |  |
| 0.125 | | 0.000125 | 6.3 | 0.003869184 | | 0.002 |  |
| 0.25 | | 0.00025 | 13 | 0.010891819 | | 0.001 |  |
| 0.5 | | 0.0005 | 25 | 0.022174651 | | 0.005 |  |
| 1 | | 0.001 | 50 | 0.080301021 | | 0.011 |  |
| 2 | | 0.002 | 100 | 0.158339144 | | 0.019 |  |
| 4 | | 0.004 | 200 | 0.350888771 | | 0.035 |  |
| 8 | | 0.008 | 400 | 0.499197278 | | 0.041 |  |
| 10 | | 0.01 | 500 | 0.656561579 | | 0.053 |  |
| **Parallel 1** |  | |  |  |  | |  |
| [S] |  | |  |  |  | |  |
| pmol/20 µL | nmol/20 µL | | nM | cleavage, % | cleavage, nM | | velocity, nM/min |
| 0.125 | 0.000125 | | 6.25 | 1.79257443 | 0.1120359 | | 0.00373453 |
| 0.25 | 0.00025 | | 12.5 | 2.49893888 | 0.31236736 | | 0.010412245 |
| 0.5 | 0.0005 | | 25 | 2.69387749 | 0.67346937 | | 0.022448979 |
| 1 | 0.001 | | 50 | 4.50410742 | 2.25205371 | | 0.075068457 |
| 2 | 0.002 | | 100 | 4.31392072 | 4.31392072 | | 0.143797357 |
| 4 | 0.004 | | 200 | 5.74320568 | 11.4864114 | | 0.382880379 |
| 8 | 0.008 | | 400 | 3.9678795 | 15.871518 | | 0.5290506 |
| 10 | 0.01 | | 500 | 4.41665225 | 22.0832612 | | 0.736108708 |
| **Parallel 2** |  | |  |  |  | |  |
| [S] |  | |  |  |  | |  |
| pmol/20 µL | nmol/20 µL | | nM | cleavage, % | cleavage, nM | | velocity, nM/min |
| 0.125 | 0.000125 | | 6.25 | 2.8094676 | 0.17559173 | | 0.005853058 |
| 0.25 | 0.00025 | | 12.5 | 2.93121148 | 0.36640143 | | 0.012213381 |
| 0.5 | 0.0005 | | 25 | 2.60832379 | 0.65208095 | | 0.021736032 |
| 1 | 0.001 | | 50 | 4.46296631 | 2.23148316 | | 0.074382772 |
| 2 | 0.002 | | 100 | 4.5827879 | 4.5827879 | | 0.152759597 |
| 4 | 0.004 | | 200 | 5.18245139 | 10.3649028 | | 0.345496759 |
| 8 | 0.008 | | 400 | 3.32809786 | 13.3123914 | | 0.443746381 |
| 10 | 0.01 | | 500 | 3.76765649 | 18.8382825 | | 0.627942748 |

C**∙**C

| **Parallel 3** |  |  |  |  |  |
| --- | --- | --- | --- | --- | --- |
| [S] |  |  |  |  |  |
| pmol/20 µL | nmol/20 µL | nM | cleavage, % | cleavage, nM | velocity, nM/min |
| 0.25 | 0.00025 | 12.5 | 2.66915127 | 0.33364391 | 0.011121464 |
| 0.5 | 0.0005 | 25 | 1.80426898 | 0.45106725 | 0.015035575 |
| 1 | 0.001 | 50 | 4.18069644 | 2.09034822 | 0.069678274 |
| 2 | 0.002 | 100 | 4.32456952 | 4.32456952 | 0.144152317 |
| 4 | 0.004 | 200 | 4.79010647 | 9.58021295 | 0.319340432 |
| 8 | 0.008 | 400 | 3.50853947 | 14.0341579 | 0.467805263 |
| 10 | 0.01 | 500 | 3.81721255 | 19.0860627 | 0.636202091 |
| **Parallel 4** |  |  |  |  |  |
| [S] |  |  |  |  |  |
| pmol/20 µL | nmol/20 µL | nM | cleavage, % | cleavage, nM | velocity, nM/min |
| 0.125 | 0.000125 | 6.25 | 2.64338077 | 0.1652113 | 0.005507043 |
| 0.25 | 0.00025 | 12.5 | 2.57010849 | 0.32126356 | 0.010708785 |
| 0.5 | 0.0005 | 25 | 2.94972145 | 0.73743036 | 0.024581012 |
| 1 | 0.001 | 50 | 5.73276026 | 2.86638013 | 0.095546004 |
| 2 | 0.002 | 100 | 5.71965823 | 5.71965823 | 0.190655274 |
| 4 | 0.004 | 200 | 5.86467229 | 11.7293446 | 0.390978153 |
| 8 | 0.008 | 400 | 3.90087785 | 15.6035114 | 0.520117046 |
| **Parallel 5** |  |  |  |  |  |
| [S] |  |  |  |  |  |
| pmol/20 µL | nmol/20 µL | nM | cleavage, % | cleavage, nM | velocity, nM/min |
| 0.125 | 0.000125 | 6.25 | 2.04061903 | 0.12753869 | 0.00425129 |
| 0.25 | 0.00025 | 12.5 | 2.40077322 | 0.30009665 | 0.010003222 |
| 0.5 | 0.0005 | 25 | 3.24859903 | 0.81214976 | 0.027071659 |
| 1 | 0.001 | 50 | 5.20977592 | 2.60488796 | 0.086829599 |
| 2 | 0.002 | 100 | 4.80993526 | 4.80993526 | 0.160331175 |
| 4 | 0.004 | 200 | 4.736222 | 9.47244401 | 0.315748134 |
| 8 | 0.008 | 400 | 4.01450325 | 16.058013 | 0.5352671 |
| 10 | 0.01 | 500 | 3.75595662 | 18.7797831 | 0.625992769 |

T**∙**T

| **[S]** | |  |  | **velocity, nM/min** | |  |
| --- | --- | --- | --- | --- | --- | --- |
| **pmol/20 µL** | | **nmol/20 µL** | **nM** | **Average** | **SD** |  |
| 0.125 | | 0.000125 | 6.25 | 0.00469438 | 0.002 |  |
| 0.25 | | 0.00025 | 12.5 | 0.00855593 | 0.005 |  |
| 0.5 | | 0.0005 | 25 | 0.02849986 | 0.012 |  |
| 1 | | 0.001 | 50 | 0.11792463 | 0.062 |  |
| 2 | | 0.002 | 100 | 0.19785082 | 0.087 |  |
| 4 | | 0.004 | 200 | 0.35025075 | 0.119 |  |
| 8 | | 0.008 | 400 | 0.4385017 | 0.186 |  |
| 10 | | 0.01 | 500 | 0.55529732 | 0.187 |  |
| **Parallel 1** |  |  |  |  | |  |
| [S] |  |  |  |  | |  |
| pmol/20 µL | nmol/20 µL | nM | cleavage, % | cleavage, nM | | velocity, nM/min |
| 0.125 | 0.000125 | 6.25 | 1.1068824 | 0.06918015 | | 0.002306005 |
| 0.25 | 0.00025 | 12.5 | 0.28041774 | 0.035052217 | | 0.001168407 |
| 0.5 | 0.0005 | 25 | 5.47289156 | 1.36822289 | | 0.04560743 |
| 1 | 0.001 | 50 | 8.18589728 | 4.092948641 | | 0.136431621 |
| 2 | 0.002 | 100 | 5.27789016 | 5.277890162 | | 0.175929672 |
| 4 | 0.004 | 200 | 6.98514598 | 13.97029196 | | 0.465676399 |
| 8 | 0.008 | 400 | 5.37908982 | 21.51635926 | | 0.717211975 |
| 10 | 0.01 | 500 | 5.14617905 | 25.73089527 | | 0.857696509 |
| **Parallel 2** |  |  |  |  | |  |
| [S] |  |  |  |  | |  |
| pmol/20 µL | nmol/20 µL | nM | cleavage, % | cleavage, nM | | velocity, nM/min |
| 0.125 | 0.000125 | 6.25 | 2.56584461 | 0.160365288 | | 0.00534551 |
| 0.25 | 0.00025 | 12.5 | 3.18491706 | 0.398114632 | | 0.013270488 |
| 0.5 | 0.0005 | 25 | 3.28159009 | 0.820397522 | | 0.027346584 |
| 1 | 0.001 | 50 | 9.29914345 | 4.649571727 | | 0.154985724 |
| 2 | 0.002 | 100 | 10.5862372 | 10.58623716 | | 0.352874572 |
| 4 | 0.004 | 200 | 7.22631024 | 14.45262048 | | 0.481754016 |
| 8 | 0.008 | 400 | 3.704393 | 14.81757199 | | 0.493919066 |
| 10 | 0.01 | 500 | 3.19221619 | 15.96108093 | | 0.532036031 |

T**∙**T

| **Parallel 3** |  |  |  |  |  |
| --- | --- | --- | --- | --- | --- |
| [S] |  |  |  |  |  |
| pmol/20 µL | nmol/20 µL | nM | cleavage, % | cleavage, nM | velocity, nM/min |
| 0.125 | 0.000125 | 6.25 | 3.31261567 | 0.207038479 | 0.006901283 |
| 0.25 | 0.00025 | 12.5 | 2.33544304 | 0.29193038 | 0.009731013 |
| 0.5 | 0.0005 | 25 | 4.0161212 | 1.004030301 | 0.033467677 |
| 1 | 0.001 | 50 | 2.53894809 | 1.269474047 | 0.042315802 |
| 2 | 0.002 | 100 | 4.65822835 | 4.658228349 | 0.155274278 |
| 4 | 0.004 | 200 | 4.25516984 | 8.510339676 | 0.283677989 |
| 8 | 0.008 | 400 | 2.39001409 | 9.560056375 | 0.318668546 |
| 10 | 0.01 | 500 | 2.40689517 | 12.03447583 | 0.401149194 |
| **Parallel 4** |  |  |  |  |  |
| [S] |  |  |  |  |  |
| pmol/20 µL | nmol/20 µL | nM | cleavage, % | cleavage, nM | velocity, nM/min |
| 0.125 | 0.000125 | 6.25 | 1.4242701 | 0.089016881 | 0.002967229 |
| 0.25 | 0.00025 | 12.5 | 1.95224019 | 0.244030024 | 0.008134334 |
| 0.5 | 0.0005 | 25 | 1.50637858 | 0.376594646 | 0.012553155 |
| 1 | 0.001 | 50 | 3.88578662 | 1.942893309 | 0.06476311 |
| 2 | 0.002 | 100 | 4.32868154 | 4.328681543 | 0.144289385 |
| 4 | 0.004 | 200 | 3.13490989 | 6.269819772 | 0.208993992 |
| 8 | 0.008 | 400 | 1.74144307 | 6.965772271 | 0.232192409 |
| 10 | 0.01 | 500 | 3.48795409 | 17.43977044 | 0.581325681 |
| **Parallel 5** |  |  |  |  |  |
| [S] |  |  |  |  |  |
| pmol/20 µL | nmol/20 µL | nM | cleavage, % | cleavage, nM | velocity, nM/min |
| 0.125 | 0.000125 | 6.25 | 2.85690444 | 0.178556527 | 0.005951884 |
| 0.25 | 0.00025 | 12.5 | 2.51409879 | 0.314262348 | 0.010475412 |
| 0.5 | 0.0005 | 25 | 2.82293719 | 0.705734298 | 0.023524477 |
| 1 | 0.001 | 50 | 11.4676148 | 5.733807408 | 0.191126914 |
| 2 | 0.002 | 100 | 4.82658639 | 4.826586393 | 0.160886213 |
| 4 | 0.004 | 200 | 4.66727013 | 9.334540255 | 0.311151342 |
| 8 | 0.008 | 400 | 3.2288738 | 12.91549521 | 0.430516507 |
| 10 | 0.01 | 500 | 2.42567501 | 12.12837506 | 0.404279169 |

m*^N^*^4^C**∙**C

| **[S]** | |  | |  | | **velocity, nM/min** | | |  |  |
| --- | --- | --- | --- | --- | --- | --- | --- | --- | --- | --- |
| **pmol/20 µL** | | **nmol/20 µL** | | **nM** | | **Average** | | **SD** |  |  |
| 0.125 | | 0.000125 | | 6.25 | | 0.00588403 | | 0.005 |  |  |
| 0.25 | | 0.00025 | | 12.5 | | 0.01316618 | | 0.003 |  |  |
| 0.5 | | 0.0005 | | 25 | | 0.03366474 | | 0.010 |  |  |
| 1 | | 0.001 | | 50 | | 0.09944813 | | 0.031 |  |  |
| 2 | | 0.002 | | 100 | | 0.21439431 | | 0.035 |  |  |
| 4 | | 0.004 | | 200 | | 0.30320116 | | 0.049 |  |  |
| 8 | | 0.008 | | 400 | | 0.35829702 | | 0.034 |  |  |
| 10 | | 0.01 | | 500 | | 0.38032715 | | 0.022 |  |  |
| **Parallel 1** |  | |  | |  | |  | | |  |
| [S] |  | |  | |  | |  | | |  |
| pmol/20 µL | nmol/20 µL | | nM | | cleavage, % | | cleavage, nM | | | velocity, nM/min |
| 0.125 | 0.000125 | | 6.25 | | 0.68559473 | | 0.04284967 | | | 0.001428322 |
| 0.25 | 0.00025 | | 12.5 | | 2.50295157 | | 0.31286895 | | | 0.010428965 |
| 0.5 | 0.0005 | | 25 | | 2.73677945 | | 0.68419486 | | | 0.022806495 |
| 1 | 0.001 | | 50 | | 7.81924203 | | 3.90962102 | | | 0.130320701 |
| 4 | 0.004 | | 200 | | 3.20835867 | | 6.41671735 | | | 0.213890578 |
| 8 | 0.008 | | 400 | | 2.31232437 | | 9.24929748 | | | 0.308309916 |
| 10 | 0.01 | | 500 | | 2.37408419 | | 11.8704209 | | | 0.395680698 |
| **Parallel 2** |  | |  | |  | |  | | |  |
| [S] |  | |  | |  | |  | | |  |
| pmol/20 µL | nmol/20 µL | | nM | | cleavage, % | | cleavage, nM | | | velocity, nM/min |
| 0.125 | 0.000125 | | 6.25 | | 0.48780942 | | 0.03048809 | | | 0.00101627 |
| 0.25 | 0.00025 | | 12.5 | | 3.40619708 | | 0.42577463 | | | 0.014192488 |
| 0.5 | 0.0005 | | 25 | | 4.01485875 | | 1.00371469 | | | 0.033457156 |
| 1 | 0.001 | | 50 | | 6.8844622 | | 3.4422311 | | | 0.114741037 |
| 2 | 0.002 | | 100 | | 7.32176158 | | 7.32176158 | | | 0.244058719 |
| 4 | 0.004 | | 200 | | 4.66325641 | | 9.32651282 | | | 0.310883761 |
| 8 | 0.008 | | 400 | | 2.79911331 | | 11.1964533 | | | 0.373215109 |
| 10 | 0.01 | | 500 | | 2.18984162 | | 10.9492081 | | | 0.364973603 |

m*^N^*^4^C**∙**C

| **Parallel 3** |  |  |  |  |  |  |
| --- | --- | --- | --- | --- | --- | --- |
| [S] |  |  |  |  |  |  |
| pmol/20 µL | nmol/20 µL | nM | cleavage, % | cleavage, nM | velocity, nM/min |  |
| 0.125 | 0.000125 | 6.25 | 5.01744693 | 0.31359043 | 0.010453014 |  |
| 0.25 | 0.00025 | 12.5 | 2.38457678 | 0.2980721 | 0.009935737 |  |
| 0.5 | 0.0005 | 25 | 5.58159116 | 1.39539779 | 0.04651326 |  |
| 1 | 0.001 | 50 | 7.37413582 | 3.68706791 | 0.122902264 |  |
| 2 | 0.002 | 100 | 7.51286935 | 7.51286935 | 0.250428978 |  |
| 4 | 0.004 | 200 | 5.15927041 | 10.3185408 | 0.34395136 |  |
| 8 | 0.008 | 400 | 2.7403464 | 10.9613856 | 0.36537952 |  |
| **Parallel 4** |  |  |  |  |  |  |
| [S] |  |  |  |  |  |  |
| pmol/20 µL | nmol/20 µL | nM | cleavage, % | cleavage, nM | velocity, nM/min |  |
| 0.125 | 0.000125 | 6.25 | 2.74272478 | 0.1714203 | 0.00571401 |  |
| 0.25 | 0.00025 | 12.5 | 3.23777188 | 0.40472148 | 0.013490716 |  |
| 0.5 | 0.0005 | 25 | 3.41768602 | 0.85442151 | 0.028480717 |  |
| 1 | 0.001 | 50 | 3.86415273 | 1.93207637 | 0.064402546 |  |
| 2 | 0.002 | 100 | 5.57879907 | 5.57879907 | 0.185959969 |  |
| 4 | 0.004 | 200 | 5.19370602 | 10.387412 | 0.346247068 |  |
| **Parallel 5** |  |  |  |  |  |  |
| [S] |  |  |  |  |  |  |
| pmol/20 µL | nmol/20 µL | nM | cleavage, % | cleavage, nM | velocity, nM/min |  |
| 0.125 | 0.000125 | 6.25 | 1.77745406 | 0.11109088 | 0.003703029 |  |
| 0.25 | 0.00025 | 12.5 | 3.07288751 | 0.38411094 | 0.012803698 |  |
| 0.5 | 0.0005 | 25 | 3.24842083 | 0.81210521 | 0.027070174 |  |
| 1 | 0.001 | 50 | 3.45623849 | 1.72811924 | 0.057603975 |  |
| 2 | 0.002 | 100 | 5.1440684 | 5.1440684 | 0.171468947 |  |
| 4 | 0.004 | 200 | 4.28525656 | 8.57051312 | 0.285683771 |  |
| 8 | 0.008 | 400 | 2.89712663 | 11.5885065 | 0.386283551 |  |
| **Parallel 6** | |  |  |  |  |  |
| [S] | |  |  |  |  |  |
| pmol/20 µL | | nmol/20 µL | nM | cleavage, % | cleavage, nM | velocity, nM/min |
| 0.125 | | 0.000125 | 6.25 | 6.2349798 | 0.38968624 | 0.012989541 |
| 0.25 | | 0.00025 | 12.5 | 4.35492097 | 0.54436512 | 0.018145504 |
| 0.5 | | 0.0005 | 25 | 5.23927744 | 1.30981936 | 0.043660645 |
| 1 | | 0.001 | 50 | 6.40309449 | 3.20154725 | 0.106718242 |
| 2 | | 0.002 | 100 | 6.60164881 | 6.60164881 | 0.22005496 |
| 4 | | 0.004 | 200 | 4.77825624 | 9.55651248 | 0.318550416 |

m^5^C**∙**C

| **[S]** | |  | |  | **velocity, nM/min** | |  |  |
| --- | --- | --- | --- | --- | --- | --- | --- | --- |
| **pmol/20 µL** | | **nmol/20 µL** | | **nM** | **Average** | **SD** |  |  |
| 0.125 | | 0.000125 | | 6.25 | 0.00958994 | 0.004 |  |  |
| 0.25 | | 0.00025 | | 12.5 | 0.01776629 | 0.011 |  |  |
| 0.5 | | 0.0005 | | 25 | 0.04719738 | 0.014 |  |  |
| 1 | | 0.001 | | 50 | 0.23964882 | 0.048 |  |  |
| 2 | | 0.002 | | 100 | 0.34090192 | 0.035 |  |  |
| 4 | | 0.004 | | 200 | 0.58481667 | 0.135 |  |  |
| 8 | | 0.008 | | 400 | 0.8705271 | 0.152 |  |  |
| 10 | | 0.01 | | 500 | 0.99184724 | 0.228 |  |  |
| **Parallel 1** | | |  |  |  |  | |  |
| [S] | | |  |  |  |  | |  |
| pmol/20 µL | | | nmol/20 µL | nM | cleavage, % | cleavage, nM | | velocity, nM/min |
| 0.125 | | | 0.000125 | 6.3 | 6.03793529 | 0.37737096 | | 0.012579032 |
| 0.25 | | | 0.00025 | 13 | 6.01645655 | 0.75205707 | | 0.025068569 |
| 0.5 | | | 0.0005 | 25 | 5.54657469 | 1.38664367 | | 0.046221456 |
| **Parallel 2** | |  |  |  |  | |  |  |
| [S] | |  |  |  |  | |  |  |
| pmol/20 µL | | nmol/20 µL | nM | cleavage, % | cleavage, nM | | velocity, nM/min |  |
| 0.125 | | 0.000125 | 6.3 | 6.12368215 | 0.38273013 | | 0.012757671 |  |
| 0.25 | | 0.00025 | 13 | 6.08408008 | 0.76051001 | | 0.025350334 |  |
| 0.5 | | 0.0005 | 25 | 6.8064735 | 1.70161838 | | 0.056720613 |  |
| 1 | | 0.001 | 50 | 12.4484971 | 6.22424853 | | 0.207474951 |  |
| 2 | | 0.002 | 100 | 10.4765396 | 10.4765396 | | 0.349217986 |  |
| 8 | | 0.008 | 400 | 8.01369178 | 32.0547671 | | 1.068492238 |  |
| **Parallel 3** | |  |  |  |  | |  |  |
| [S] | |  |  |  |  | |  |  |
| pmol/20 µL | | nmol/20 µL | nM | cleavage, % | cleavage, nM | | velocity, nM/min |  |
| 0.125 | | 0.000125 | 6.3 | 6.21594823 | 0.38849676 | | 0.012949892 |  |
| 0.25 | | 0.00025 | 13 | 6.04162225 | 0.75520278 | | 0.025173426 |  |
| 0.5 | | 0.0005 | 25 | 8.76926433 | 2.19231608 | | 0.073077203 |  |
| 1 | | 0.001 | 50 | 12.9842162 | 6.49210809 | | 0.216403603 |  |
| **Parallel 4** | |  |  |  |  | |  |  |
| [S] | |  |  |  |  | |  |  |
| pmol/20 µL | | nmol/20 µL | nM | cleavage, % | cleavage, nM | | velocity, nM/min |  |
| 0.125 | | 0.000125 | 6.3 | 6.21948334 | 0.38871771 | | 0.012957257 |  |
| 0.25 | | 0.00025 | 13 | 6.9807 | 0.8725875 | | 0.02908625 |  |
| 0.5 | | 0.0005 | 25 | 7.09959658 | 1.77489914 | | 0.059163305 |  |
| 2 | | 0.002 | 100 | 11.8428765 | 11.8428765 | | 0.39476255 |  |
| 8 | | 0.008 | 400 | 6.73302211 | 26.9320885 | | 0.897736282 |  |

m^5^C**∙**C

| **Parallel 5** |  |  |  |  |  |
| --- | --- | --- | --- | --- | --- |
| [S] |  |  |  |  |  |
| pmol/20 µL | nmol/20 µL | nM | cleavage, % | cleavage, nM | velocity, nM/min |
| 0.5 | 0.0005 | 25 | 6.24730183 | 1.561825458 | 0.052060849 |
| 1 | 0.001 | 50 | 17.704074 | 8.852037019 | 0.295067901 |
| 4 | 0.004 | 200 | 10.7396032 | 21.47920642 | 0.715973547 |
| 8 | 0.008 | 400 | 6.03938837 | 24.15755349 | 0.805251783 |
| **Parallel 6** |  |  |  |  |  |
| [S] |  |  |  |  |  |
| pmol/20 µL | nmol/20 µL | nM | cleavage, % | cleavage, nM | velocity, nM/min |
| 0.125 | 0.000125 | 6.25 | 2.55086279 | 0.159428924 | 0.005314297 |
| 0.5 | 0.0005 | 25 | 2.57742347 | 0.644355868 | 0.021478529 |
| 2 | 0.002 | 100 | 10.2064673 | 10.20646734 | 0.340215578 |
| 4 | 0.004 | 200 | 8.87937974 | 17.75875948 | 0.591958649 |
| 8 | 0.008 | 400 | 5.32971059 | 21.31884237 | 0.710628079 |
| 10 | 0.01 | 500 | 4.9969523 | 24.98476148 | 0.832825383 |
| **Parallel 7** |  |  |  |  |  |
| [S] |  |  |  |  |  |
| pmol/20 µL | nmol/20 µL | nM | cleavage, % | cleavage, nM | velocity, nM/min |
| 0.125 | 0.000125 | 6.25 | 4.38426905 | 0.274016815 | 0.009133894 |
| 0.25 | 0.00025 | 12.5 | 5.77836549 | 0.722295686 | 0.024076523 |
| 0.5 | 0.0005 | 25 | 5.77570667 | 1.443926667 | 0.048130889 |
| 2 | 0.002 | 100 | 9.11419922 | 9.114199216 | 0.303806641 |
| 4 | 0.004 | 200 | 6.69776727 | 13.39553453 | 0.446517818 |
| 10 | 0.01 | 500 | 7.5217418 | 37.60870902 | 1.253623634 |
| **Parallel 8** |  |  |  |  |  |
| [S] |  |  |  |  |  |
| pmol/20 µL | nmol/20 µL | nM | cleavage, % | cleavage, nM | velocity, nM/min |
| 0.125 | 0.000125 | 6.25 | 5.26066754 | 0.328791721 | 0.010959724 |
| 0.25 | 0.00025 | 12.5 | 4.04001712 | 0.50500214 | 0.016833405 |
| 0.5 | 0.0005 | 25 | 5.67396458 | 1.418491146 | 0.047283038 |
| 2 | 0.002 | 100 | 9.4952053 | 9.495205297 | 0.316506843 |
| 10 | 0.01 | 500 | 5.33455624 | 26.6727812 | 0.889092707 |

m^5^C**∙**C

| **Parallel 9** |  |  |  |  |  |
| --- | --- | --- | --- | --- | --- |
| [S] |  |  |  |  |  |
| pmol/20 µL | nmol/20 µL | nM | cleavage, % | cleavage, nM | velocity, nM/min |
| 0.125 | 0.000125 | 6.25 | 4.63567961 | 0.289729976 | 0.009657666 |
| 0.25 | 0.00025 | 12.5 | 3.43393671 | 0.429242088 | 0.01430807 |
| 0.5 | 0.0005 | 25 | 5.58244164 | 1.395610409 | 0.046520347 |

m^5^C**∙**T

| **[S]** |  |  | **velocity, nM/min** | | | |  |  |
| --- | --- | --- | --- | --- | --- | --- | --- | --- |
| **pmol/20 µL** | **nmol/20 µL** | **nM** | **Average** | | **SD** | |  |  |
| 0.125 | 0.000125 | 6.25 | 0.00320205 | | 0.001 | |  |  |
| 0.25 | 0.00025 | 12.5 | 0.00590743 | | 0.003 | |  |  |
| 0.5 | 0.0005 | 25 | 0.01748805 | | 0.007 | |  |  |
| 1 | 0.001 | 50 | 0.07295418 | | 0.014 | |  |  |
| 2 | 0.002 | 100 | 0.07831777 | | 0.036 | |  |  |
| 4 | 0.004 | 200 | 0.20669422 | | 0.013 | |  |  |
| 8 | 0.008 | 400 | 0.33368562 | | 0.049 | |  |  |
| 10 | 0.01 | 500 | 0.39332682 | | 0.018 | |  |  |
| **Parallel 1** |  |  | |  | |  | |  |
| [S] |  |  | |  | |  | |  |
| pmol/20 µL | nmol/20 µL | nM | | cleavage, % | | cleavage, nM | | velocity, nM/min |
| 0.125 | 0.000125 | 6.3 | | 1.27781363 | | 0.079863352 | | 0.002662112 |
| 0.25 | 0.00025 | 13 | | 1.39193987 | | 0.173992484 | | 0.005799749 |
| 0.5 | 0.0005 | 25 | | 3.22593563 | | 0.806483907 | | 0.026882797 |
| 4 | 0.004 | 200 | | 3.17791093 | | 6.355821858 | | 0.211860729 |
| 8 | 0.008 | 400 | | 2.24139553 | | 8.965582129 | | 0.298852738 |
| 10 | 0.01 | 500 | | 2.24139553 | | 11.20697766 | | 0.373565922 |
| **Parallel 2** |  |  | |  | |  | |  |
| [S] |  |  | |  | |  | |  |
| pmol/20 µL | nmol/20 µL | nM | | cleavage, % | | cleavage, nM | | velocity, nM/min |
| 0.125 | 0.000125 | 6.3 | | 1.38329042 | | 0.086455651 | | 0.002881855 |
| 0.25 | 0.00025 | 13 | | 1.28112011 | | 0.160140014 | | 0.005338 |
| 0.5 | 0.0005 | 25 | | 1.46297137 | | 0.365742844 | | 0.012191428 |
| 1 | 0.001 | 50 | | 4.23424902 | | 2.117124509 | | 0.070570817 |
| 4 | 0.004 | 200 | | 3.24393439 | | 6.487868786 | | 0.216262293 |
| 8 | 0.008 | 400 | | 2.76388873 | | 11.0555549 | | 0.368518497 |
| 10 | 0.01 | 500 | | 2.46073549 | | 12.30367744 | | 0.410122581 |

m^5^C**∙**T

| **Parallel 3** |  |  |  |  |  |
| --- | --- | --- | --- | --- | --- |
| [S] |  |  |  |  |  |
| pmol/20 µL | nmol/20 µL | nM | cleavage, % | cleavage, nM | velocity, nM/min |
| 0.125 | 0.000125 | 6.3 | 1.86695982 | 0.116684989 | 0.0038895 |
| 0.25 | 0.00025 | 13 | 1.96173657 | 0.245217071 | 0.008173902 |
| 0.5 | 0.0005 | 25 | 2.63137177 | 0.657842943 | 0.021928098 |
| 1 | 0.001 | 50 | 5.24419233 | 2.622096167 | 0.087403206 |
| 2 | 0.002 | 100 | 3.3987516 | 3.398751601 | 0.11329172 |
| 4 | 0.004 | 200 | 2.87939436 | 5.758788729 | 0.191959624 |
| 10 | 0.01 | 500 | 2.37775181 | 11.88875905 | 0.396291968 |
| **Parallel 4** |  |  |  |  |  |
| [S] |  |  |  |  |  |
| pmol/20 µL | nmol/20 µL | nM | cleavage, % | cleavage, nM | velocity, nM/min |
| 0.125 | 0.000125 | 6.3 | 1.45666558 | 0.0910416 | 0.00303472 |
| **Parallel 5** |  |  |  |  |  |
| [S] |  |  |  |  |  |
| pmol/20 µL | nmol/20 µL | nM | cleavage, % | cleavage, nM | velocity, nM/min |
| 0.25 | 0.00025 | 13 | 1.9726752 | 0.2465844 | 0.00821948 |
| 0.5 | 0.0005 | 25 | 1.56578544 | 0.39144636 | 0.013048212 |
| **Parallel 6** |  |  |  |  |  |
| [S] |  |  |  |  |  |
| pmol/20 µL | nmol/20 µL | nM | cleavage, % | cleavage, nM | velocity, nM/min |
| 1 | 0.001 | 50 | 3.31988195 | 1.65994098 | 0.055331366 |
| 2 | 0.002 | 100 | 1.24564966 | 1.24564966 | 0.041521655 |
| **Parallel 7** |  |  |  |  |  |
| [S] |  |  |  |  |  |
| pmol/20 µL | nmol/20 µL | nM | cleavage, % | cleavage, nM | velocity, nM/min |
| 0.5 | 0.0005 | 25 | 1.60676868 | 0.40169217 | 0.013389739 |
| **Parallel 8** |  |  |  |  |  |
| [S] |  |  |  |  |  |
| pmol/20 µL | nmol/20 µL | nM | cleavage, % | cleavage, nM | velocity, nM/min |
| 0.125 | 0.000125 | 6.3 | 1.70018033 | 0.10626127 | 0.003542042 |
| 0.25 | 0.00025 | 13 | 0.48144193 | 0.06018024 | 0.002006008 |
| 1 | 0.001 | 50 | 4.71068107 | 2.35534054 | 0.078511351 |
| 2 | 0.002 | 100 | 2.40419804 | 2.40419804 | 0.080139935 |

m*^N^*^4,5^C**∙**C

| **[S]** | |  |  | **velocity, nM/min** | |  |
| --- | --- | --- | --- | --- | --- | --- |
| **pmol/20 µL** | | **nmol/20 µL** | **nM** | **Average** | **SD** |  |
| 0.125 | | 0.000125 | 6.25 | 0.022655 | 0.009 |  |
| 0.25 | | 0.00025 | 12.5 | 0.035549 | 0.009 |  |
| 0.5 | | 0.0005 | 25 | 0.09147 | 0.012 |  |
| 1 | | 0.001 | 50 | 0.192025 | 0.021 |  |
| 2 | | 0.002 | 100 | 0.36177 | 0.035 |  |
| 4 | | 0.004 | 200 | 0.745269 | 0.081 |  |
| 8 | | 0.008 | 400 | 1.345078 | 0.087 |  |
| 10 | | 0.01 | 500 | 1.577857 | 0.075 |  |
| **Parallel 1** |  |  |  |  | |  |
| [S] |  |  |  |  | |  |
| pmol/20 µL | nmol/20 µL | nM | cleavage, % | cleavage, nM | | velocity, nM/min |
| 0.125 | 0.000125 | 6.25 | 20.6246322 | 1.289039514 | | 0.042967984 |
| 0.25 | 0.00025 | 12.5 | 9.42829152 | 1.17853644 | | 0.039284548 |
| 0.5 | 0.0005 | 25 | 11.8816539 | 2.970413472 | | 0.099013782 |
| 1 | 0.001 | 50 | 10.8990104 | 5.44950519 | | 0.181650173 |
| 2 | 0.002 | 100 | 11.4945856 | 11.49458564 | | 0.383152855 |
| **Parallel 2** |  |  |  |  | |  |
| [S] |  |  |  |  | |  |
| pmol/20 µL | nmol/20 µL | nM | cleavage, % | cleavage, nM | | velocity, nM/min |
| 4 | 0.004 | 200 | 12.1358137 | 24.27162739 | | 0.809054246 |
| **Parallel 3** |  |  |  |  | |  |
| [S] |  |  |  |  | |  |
| pmol/20 µL | nmol/20 µL | nM | cleavage, % | cleavage, nM | | velocity, nM/min |
| 0.125 | 0.000125 | 6.25 | 6.83440019 | 0.427150012 | | 0.014238334 |
| 0.25 | 0.00025 | 12.5 | 5.98973135 | 0.748716419 | | 0.024957214 |
| 0.5 | 0.0005 | 25 | 9.14709977 | 2.286774942 | | 0.076225831 |
| 4 | 0.004 | 200 | 9.14888265 | 18.29776529 | | 0.60992551 |
| **Parallel 4** |  |  |  |  | |  |
| [S] |  |  |  |  | |  |
| pmol/20 µL | nmol/20 µL | nM | cleavage, % | cleavage, nM | | velocity, nM/min |
| 0.25 | 0.00025 | 12.5 | 9.5381927 | 1.192274087 | | 0.03974247 |
| 2 | 0.002 | 100 | 10.4935877 | 10.49358773 | | 0.349786258 |
| 10 | 0.01 | 500 | 8.81979182 | 44.09895911 | | 1.469965304 |
| **Parallel 5** |  |  |  |  | |  |
| [S] |  |  |  |  | |  |
| pmol/20 µL | nmol/20 µL | nM | cleavage, % | cleavage, nM | | velocity, nM/min |
| 2 | 0.002 | 100 | 9.72280072 | 9.722800721 | | 0.324093357 |
| 4 | 0.004 | 200 | 12.8344475 | 25.66889509 | | 0.855629836 |

m*^N^*^4,5^C**∙**C

| **Parallel 6** |  |  |  | |  | |  | |
| --- | --- | --- | --- | --- | --- | --- | --- | --- |
| [S] |  |  |  | |  | |  | |
| pmol/20 µL | nmol/20 µL | nM | cleavage, % | | cleavage, nM | | velocity, nM/min | |
| 0.125 | 0.000125 | 6.25 | 11.5058442 | | 0.719115262 | | 0.023970509 | |
| 0.25 | 0.00025 | 12.5 | 6.898019 | | 0.862252375 | | 0.028741746 | |
| 0.5 | 0.0005 | 25 | 11.6574416 | | 2.914360412 | | 0.097145347 | |
| 1 | 0.001 | 50 | 14.4334492 | | 7.216724595 | | 0.240557487 | |
| 4 | 0.004 | 200 | 11.0137898 | | 22.02757969 | | 0.734252656 | |
| 8 | 0.008 | 400 | 10.2405432 | | 40.96217297 | | 1.365405766 | |
| 10 | 0.01 | 500 | 9.68143086 | | 48.4071543 | | 1.61357181 | |
| **Parallel 7** |  |  |  | |  | |  | |
| [S] |  |  |  | |  | |  | |
| pmol/20 µL | nmol/20 µL | nM | cleavage, % | | cleavage, nM | | velocity, nM/min | |
| 0.125 | 0.000125 | 6.25 | 5.73405383 | | 0.358378364 | | 0.011945945 | |
| 0.5 | 0.0005 | 25 | 9.73589007 | | 2.433972518 | | 0.081132417 | |
| 1 | 0.001 | 50 | 9.80533907 | | 4.902669536 | | 0.163422318 | |
| 2 | 0.002 | 100 | 12.6607867 | | 12.66078669 | | 0.422026223 | |
| 4 | 0.004 | 200 | 11.7486116 | | 23.49722329 | | 0.783240776 | |
| 8 | 0.008 | 400 | 10.25909 | | 41.03636011 | | 1.36787867 | |
| 10 | 0.01 | 500 | 9.53137097 | | 47.65685483 | | 1.588561828 | |
| **Parallel 8** |  |  |  | |  | |  | |
| [S] |  |  |  | |  | |  | |
| pmol/20 µL | nmol/20 µL | nM | cleavage, % | | cleavage, nM | | velocity, nM/min | |
| 0.125 | 0.000125 | 6.25 | 7.69596773 | | 0.480997983 | | 0.016033266 | |
| 0.25 | 0.00025 | 12.5 | 5.9724733 | | 0.746559163 | | 0.024885305 | |
| 0.5 | 0.0005 | 25 | 9.62197106 | | 2.405492766 | | 0.080183092 | |
| 1 | 0.001 | 50 | 10.9253073 | | 5.462653665 | | 0.182088456 | |
| 2 | 0.002 | 100 | 10.6169803 | | 10.6169803 | | 0.353899343 | |
| **Parallel 9** |  |  | |  | |  | |  |
| [S] |  |  | |  | |  | |  |
| pmol/20 µL | nmol/20 µL | nM | | cleavage, % | | cleavage, nM | | velocity, nM/min |
| 0.125 | 0.000125 | 6.25 | | 9.88280185 | | 0.617675116 | | 0.020589171 |
| 0.5 | 0.0005 | 25 | | 9.99232844 | | 2.498082109 | | 0.083269404 |
| 1 | 0.001 | 50 | | 11.5562706 | | 5.778135294 | | 0.19260451 |
| 2 | 0.002 | 100 | | 10.1298463 | | 10.1298463 | | 0.337661543 |

m*^N^*^4,5^C**∙**C

| **Parallel 10** |  |  |  |  |  |
| --- | --- | --- | --- | --- | --- |
| [S] |  |  |  |  |  |
| pmol/20 µL | nmol/20 µL | nM | cleavage, % | cleavage, nM | velocity, nM/min |
| 0.125 | 0.000125 | 6.25 | 16.2156543 | 1.013478397 | 0.033782613 |
| 0.25 | 0.00025 | 12.5 | 8.06015401 | 1.007519251 | 0.033583975 |
| 0.5 | 0.0005 | 25 | 12.3148119 | 3.078702979 | 0.102623433 |
| 1 | 0.001 | 50 | 11.3451908 | 5.672595422 | 0.189086514 |
| 8 | 0.008 | 400 | 9.15864454 | 36.63457817 | 1.221152606 |
| **Parallel 11** |  |  |  |  |  |
| [S] |  |  |  |  |  |
| pmol/20 µL | nmol/20 µL | nM | cleavage, % | cleavage, nM | velocity, nM/min |
| 0.125 | 0.000125 | 6.25 | 9.71903733 | 0.607439833 | 0.020247994 |
| 0.25 | 0.00025 | 12.5 | 7.92028284 | 0.990035355 | 0.033001178 |
| 0.5 | 0.0005 | 25 | 9.93457215 | 2.483643037 | 0.082788101 |
| 1 | 0.001 | 50 | 11.4532718 | 5.726635887 | 0.190887863 |
| **Parallel 12** |  |  |  |  |  |
| [S] |  |  |  |  |  |
| pmol/20 µL | nmol/20 µL | nM | cleavage, % | cleavage, nM | velocity, nM/min |
| 0.125 | 0.000125 | 6.25 | 9.20356669 | 0.575222918 | 0.019174097 |
| 0.25 | 0.00025 | 12.5 | 10.0059893 | 1.250748668 | 0.041691622 |
| 0.5 | 0.0005 | 25 | 13.4038724 | 3.350968098 | 0.111698937 |
| 1 | 0.001 | 50 | 11.1573953 | 5.578697643 | 0.185956588 |
| 4 | 0.004 | 200 | 10.9801055 | 21.96021098 | 0.732007033 |
| 8 | 0.008 | 400 | 10.6940572 | 42.77622883 | 1.425874294 |
| **Parallel 13** |  |  |  |  |  |
| [S] |  |  |  |  |  |
| pmol/20 µL | nmol/20 µL | nM | cleavage, % | cleavage, nM | velocity, nM/min |
| 0.125 | 0.000125 | 6.25 | 11.3286056 | 0.70803785 | 0.023601262 |
| 0.25 | 0.00025 | 12.5 | 12.9724238 | 1.621552981 | 0.054051766 |
| 0.5 | 0.0005 | 25 | 12.0747015 | 3.018675379 | 0.100622513 |
| 1 | 0.001 | 50 | 12.1185019 | 6.059250953 | 0.201975032 |
| 4 | 0.004 | 200 | 10.3916196 | 20.78323919 | 0.69277464 |
| 10 | 0.01 | 500 | 9.83596408 | 49.1798204 | 1.639327347 |

dHT**∙**A

| **[S]** |  |  | **velocity, nM/min** | | | |  |  |
| --- | --- | --- | --- | --- | --- | --- | --- | --- |
| **pmol/20 µL** | **nmol/20 µL** | **nM** | **Average** | | **SD** | |  |  |
| 0.125 | 0.000125 | 6.3 | 0.011469 | | 0.001 | |  |  |
| 0.25 | 0.00025 | 13 | 0.019985 | | 0.004 | |  |  |
| 0.5 | 0.0005 | 25 | 0.072588 | | 0.002 | |  |  |
| 1 | 0.001 | 50 | 0.238578 | | 0.035 | |  |  |
| 2 | 0.002 | 100 | 0.447958 | | 0.057 | |  |  |
| 4 | 0.004 | 200 | 0.714321 | | 0.083 | |  |  |
| 8 | 0.008 | 400 | 1.372965 | | 0.543 | |  |  |
| 10 | 0.01 | 500 | 1.336637 | | 0.320 | |  |  |
| **Parallel 1** |  |  | |  | |  | |  |
| [S] |  |  | |  | |  | |  |
| pmol/20 µL | nmol/20 µL | nM | | cleavage, % | | cleavage, nM | | velocity, nM/min |
| 0.125 | 0.000125 | 6.3 | | 5.9314021 | | 0.37071263 | | 0.012357088 |
| 0.25 | 0.00025 | 13 | | 6.04388155 | | 0.75548519 | | 0.02518284 |
| 0.5 | 0.0005 | 25 | | 8.41113952 | | 2.10278488 | | 0.070092829 |
| 1 | 0.001 | 50 | | 16.2076376 | | 8.10381879 | | 0.270127293 |
| 2 | 0.002 | 100 | | 15.2681909 | | 15.2681909 | | 0.508939697 |
| **Parallel 2** |  |  | |  | |  | |  |
| [S] |  |  | |  | |  | |  |
| pmol/20 µL | nmol/20 µL | nM | | cleavage, % | | cleavage, nM | | velocity, nM/min |
| 0.125 | 0.000125 | 6.3 | | 5.24185559 | | 0.32761597 | | 0.010920532 |
| 0.25 | 0.00025 | 13 | | 4.74199506 | | 0.59274938 | | 0.019758313 |
| 0.5 | 0.0005 | 25 | | 8.95347883 | | 2.23836971 | | 0.074612324 |
| 1 | 0.001 | 50 | | 12.0603642 | | 6.03018212 | | 0.201006071 |
| 2 | 0.002 | 100 | | 11.8818553 | | 11.8818553 | | 0.396061843 |
| **Parallel 3** |  |  | |  | |  | |  |
| [S] |  |  | |  | |  | |  |
| pmol/20 µL | nmol/20 µL | nM | | cleavage, % | | cleavage, nM | | velocity, nM/min |
| 0.125 | 0.000125 | 6.3 | | 5.16638223 | | 0.32289889 | | 0.010763296 |
| 0.25 | 0.00025 | 13 | | 3.83705581 | | 0.47963198 | | 0.015987733 |
| 0.5 | 0.0005 | 25 | | 8.76696112 | | 2.19174028 | | 0.073058009 |
| 4 | 0.004 | 200 | | 9.83749898 | | 19.674998 | | 0.655833265 |
| 8 | 0.008 | 400 | | 13.1745512 | | 52.6982049 | | 1.75660683 |
| 10 | 0.01 | 500 | | 9.37825025 | | 46.8912512 | | 1.563041708 |

dHT**∙**A

| **Parallel 4** |  |  |  |  |  |
| --- | --- | --- | --- | --- | --- |
| [S] |  |  |  |  |  |
| pmol/20 µL | nmol/20 µL | nM | cleavage, % | cleavage, nM | velocity, nM/min |
| 0.125 | 0.000125 | 6.3 | 5.67991401 | 0.35499463 | 0.011833154 |
| 0.25 | 0.00025 | 13 | 4.56229958 | 0.57028745 | 0.019009582 |
| 1 | 0.001 | 50 | 14.6760594 | 7.33802971 | 0.24460099 |
| 2 | 0.002 | 100 | 13.1661674 | 13.1661674 | 0.438872247 |
| 4 | 0.004 | 200 | 11.592127 | 23.184254 | 0.772808466 |
| 8 | 0.008 | 400 | 7.41992063 | 29.6796825 | 0.98932275 |
| 10 | 0.01 | 500 | 6.66139764 | 33.3069882 | 1.11023294 |

Tg**∙**A

| **[S]** |  |  | **velocity, nM/min** | | |  |  |
| --- | --- | --- | --- | --- | --- | --- | --- |
| **pmol/20 µL** | **nmol/20 µL** | **nM** | **Average** | | **SD** |  |  |
| 0.125 | 0.000125 | 6.3 | 0.00609822 | | 0.002 |  |  |
| 0.25 | 0.00025 | 13 | 0.00978512 | | 0.002 |  |  |
| 0.5 | 0.0005 | 25 | 0.02347 | | 0.009 |  |  |
| 1 | 0.001 | 50 | 0.06387038 | | 0.024 |  |  |
| 2 | 0.002 | 100 | 0.26143446 | | 0.048 |  |  |
| 4 | 0.004 | 200 | 0.54402537 | | 0.343 |  |  |
| 8 | 0.008 | 400 | 0.90534984 | | 0.066 |  |  |
| 10 | 0.01 | 500 | 0.70696586 | | 0.360 |  |  |
| **Parallel 1** |  |  | |  |  | |  |
| [S] |  |  | |  |  | |  |
| pmol/20 µL | nmol/20 µL | nM | | cleavage, % | cleavage, nM | | velocity, nM/min |
| 0.125 | 0.000125 | 6.25 | | 4.27831638 | 0.26739477 | | 0.008913159 |
| 0.25 | 0.00025 | 12.5 | | 1.93600888 | 0.24200111 | | 0.008066704 |
| 0.5 | 0.0005 | 25 | | 2.68308912 | 0.67077228 | | 0.022359076 |
| 1 | 0.001 | 50 | | 2.89433417 | 1.44716708 | | 0.048238903 |
| 4 | 0.004 | 200 | | 14.027027 | 28.054054 | | 0.935135134 |
| 8 | 0.008 | 400 | | 6.90933087 | 27.6373235 | | 0.921244116 |
| 10 | 0.01 | 500 | | 5.76994017 | 28.8497009 | | 0.961656695 |
| **Parallel 2** |  |  | |  |  | |  |
| [S] |  |  | |  |  | |  |
| pmol/20 µL | nmol/20 µL | nM | | cleavage, % | cleavage, nM | | velocity, nM/min |
| 0.125 | 0.000125 | 6.25 | | 2.72255758 | 0.17015985 | | 0.005671995 |
| 0.25 | 0.00025 | 12.5 | | 1.94714761 | 0.24339345 | | 0.008113115 |
| 2 | 0.002 | 100 | | 8.52523769 | 8.52523769 | | 0.28417459 |
| 8 | 0.008 | 400 | | 7.21659139 | 28.8663656 | | 0.962212185 |

Tg**∙**A

| **Parallel 3** |  |  |  |  |  |
| --- | --- | --- | --- | --- | --- |
| [S] |  |  |  |  |  |
| pmol/20 µL | nmol/20 µL | nM | cleavage, % | cleavage, nM | velocity, nM/min |
| 0.125 | 0.000125 | 6.25 | 1.66700804 | 0.104188 | 0.003472933 |
| 0.25 | 0.00025 | 12.5 | 2.45036686 | 0.30629586 | 0.010209862 |
| 0.5 | 0.0005 | 25 | 1.84791903 | 0.46197976 | 0.015399325 |
| 1 | 0.001 | 50 | 3.13427389 | 1.56713695 | 0.052237898 |
| 2 | 0.002 | 100 | 6.17793185 | 6.17793185 | 0.205931062 |
| 4 | 0.004 | 200 | 4.44109491 | 8.88218981 | 0.296072994 |
| 10 | 0.01 | 500 | 2.71365013 | 13.5682506 | 0.452275021 |
| **Parallel 4** |  |  |  |  |  |
| [S] |  |  |  |  |  |
| pmol/20 µL | nmol/20 µL | nM | cleavage, % | cleavage, nM | velocity, nM/min |
| 0.125 | 0.000125 | 6.25 | 3.04070144 | 0.19004384 | 0.006334795 |
| 0.25 | 0.00025 | 12.5 | 3.06019296 | 0.38252412 | 0.012750804 |
| 0.5 | 0.0005 | 25 | 3.91819032 | 0.97954758 | 0.032651586 |
| 1 | 0.001 | 50 | 5.46805947 | 2.73402974 | 0.091134325 |
| 2 | 0.002 | 100 | 8.82593171 | 8.82593171 | 0.294197724 |
| 4 | 0.004 | 200 | 6.01301993 | 12.0260399 | 0.400867995 |
| 8 | 0.008 | 400 | 6.24444905 | 24.9777962 | 0.832593207 |

oxo^8^G**∙**C

| **[S]** |  |  | **velocity, nM/min** | |
| --- | --- | --- | --- | --- |
| **pmol/20 µL** | **nmol/20 µL** | **nM** | **Average** | **SD** |
| 0.125 | 0.000125 | 6.3 | 0.025523 | 0.002 |
| 0.25 | 0.00025 | 13 | 0.054638 | 0.006 |
| 0.5 | 0.0005 | 25 | 0.123794 | 0.043 |
| 1 | 0.001 | 50 | 0.303895 | 0.062 |
| 2 | 0.002 | 100 | 0.609011 | 0.141 |
| 4 | 0.004 | 200 | 0.930244 | 0.059 |
| 8 | 0.008 | 400 | 1.814631 | 0.052 |
| 10 | 0.01 | 500 | 1.868644 | 0.334 |

oxo^8^G**∙**C

| **Parallel 1** |  |  |  |  |  |
| --- | --- | --- | --- | --- | --- |
| [S] |  |  |  |  |  |
| pmol/20 µL | nmol/20 µL | nM | cleavage, % | cleavage, nM | velocity, nM/min |
| 0.125 | 0.000125 | 6.25 | 13.0523346 | 0.815770911 | 0.027192364 |
| 0.25 | 0.00025 | 12.5 | 12.6280527 | 1.578506586 | 0.052616886 |
| 0.5 | 0.0005 | 25 | 13.4560879 | 3.364021977 | 0.112134066 |
| 1 | 0.001 | 50 | 18.5534867 | 9.276743351 | 0.309224778 |
| 2 | 0.002 | 100 | 19.4762148 | 19.47621484 | 0.649207161 |
| 4 | 0.004 | 200 | 13.2237992 | 26.44759835 | 0.881586612 |
| 8 | 0.008 | 400 | 13.8859741 | 55.54389655 | 1.851463218 |
| 10 | 0.01 | 500 | 9.79474772 | 48.97373861 | 1.632457954 |
| **Parallel 2** |  |  |  |  |  |
| [S] |  |  |  |  |  |
| pmol/20 µL | nmol/20 µL | nM | cleavage, % | cleavage, nM | velocity, nM/min |
| 0.125 | 0.000125 | 6.25 | 13.0528397 | 0.81580248 | 0.027193416 |
| 0.25 | 0.00025 | 12.5 | 11.3835946 | 1.422949331 | 0.047431644 |
| 0.5 | 0.0005 | 25 | 8.66016433 | 2.165041084 | 0.072168036 |
| 1 | 0.001 | 50 | 22.591127 | 11.29556351 | 0.376518784 |
| 2 | 0.002 | 100 | 10.8212767 | 10.82127669 | 0.360709223 |
| 4 | 0.004 | 200 | 13.7034821 | 27.40696411 | 0.91356547 |
| 8 | 0.008 | 400 | 13.3334835 | 53.33393393 | 1.777797798 |
| 10 | 0.01 | 500 | 12.6289797 | 63.14489868 | 2.104829956 |
| **Parallel 3** |  |  |  |  |  |
| [S] |  |  |  |  |  |
| pmol/20 µL | nmol/20 µL | nM | cleavage, % | cleavage, nM | velocity, nM/min |
| 0.125 | 0.000125 | 6.25 | 10.8259846 | 0.676624038 | 0.022554135 |
| 0.5 | 0.0005 | 25 | 11.7172474 | 2.929311854 | 0.097643728 |
| 1 | 0.001 | 50 | 17.9593075 | 8.979653744 | 0.299321791 |
| 2 | 0.002 | 100 | 20.8126575 | 20.81265753 | 0.693755251 |
| **Parallel 4** |  |  |  |  |  |
| [S] |  |  |  |  |  |
| pmol/20 µL | nmol/20 µL | nM | cleavage, % | cleavage, nM | velocity, nM/min |
| 0.125 | 0.000125 | 6.25 | 12.9767789 | 0.811048684 | 0.027034956 |
| 0.25 | 0.00025 | 12.5 | 14.4727865 | 1.809098316 | 0.060303277 |
| 0.5 | 0.0005 | 25 | 20.8426125 | 5.210653124 | 0.173688437 |
| 1 | 0.001 | 50 | 19.6218129 | 9.810906471 | 0.327030216 |
| 2 | 0.002 | 100 | 19.3645716 | 19.36457158 | 0.645485719 |
| 4 | 0.004 | 200 | 14.9337044 | 29.86740889 | 0.995580296 |

oxo^8^G**∙**C

| **Parallel 5** |  |  |  |  |  |
| --- | --- | --- | --- | --- | --- |
| [S] |  |  |  |  |  |
| pmol/20 µL | nmol/20 µL | nM | cleavage, % | cleavage, nM | velocity, nM/min |
| 0.125 | 0.000125 | 6.25 | 11.3479594 | 0.709247465 | 0.023641582 |
| 0.25 | 0.00025 | 12.5 | 13.9679752 | 1.745996905 | 0.058199897 |
| 0.5 | 0.0005 | 25 | 19.6000676 | 4.900016897 | 0.163333897 |
| 1 | 0.001 | 50 | 12.4428639 | 6.221431969 | 0.207381066 |
| 2 | 0.002 | 100 | 20.8769467 | 20.87694669 | 0.695898223 |

# Supplementary Figures and Tables

## Supplementary Figures

**
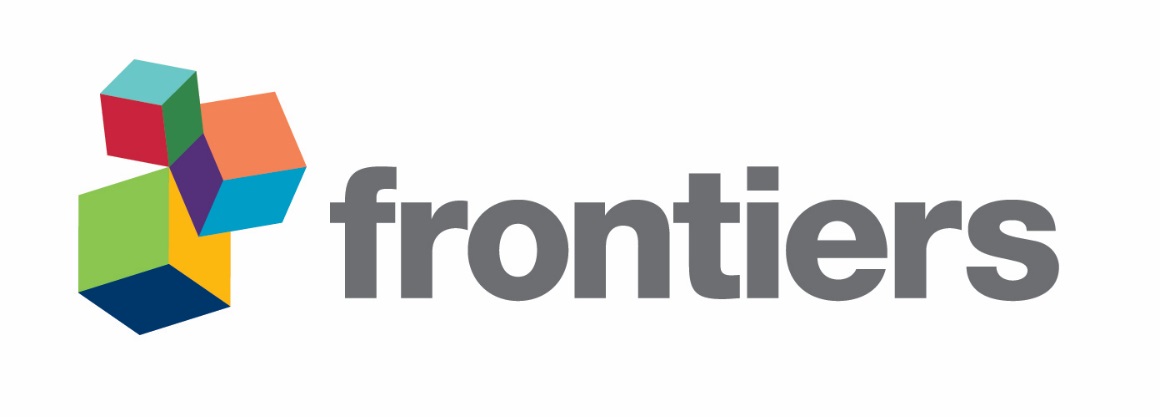
**

**Supplementary Figure 1.** Glycosylase activity assay (see “Materials and Methods”). Abbreviation: nt, nucleotides. The 5´-label and strand are indicated in magenta; see **Figure 1A** for explanation of other colors.

**Supplementary Figure 2.** Proposed steps of the BER pathway for C**∙**C in DNA. Both the base excision (step 1) and the AP site incision (step 2) are consecutively performed by the bi-functional DNA glycosylase Fpg as reported here, leaving behind a DNA polymerase blocking 3´-phosphate remnant that must be removed by a phosphatase (step 3), which may be XthA or Nfo (Doetsch and Cunningham, 1990) (**Figure 3**). The cleaned one-nucleotide-gap in DNA is now ready for the insertion of the correct dGMP (step 4) by the repair DNA polymerase I (PolA) (Patel et al., 2001) followed by nick-sealing (step 5) by DNA ligase (LigA) (Chauleau and Shuman, 2016). The residues that are removed and are the result of replacement, and their corresponding reaction arrows and enzymes, are indicated in red and green respectively. The other mismatched C is indicated in blue. Abbreviations: dR, deoxyribose; P, phosphate.

**Supplementary Figure 3.** Time dependency and opposite base-dependent kinetics of Fpg for methylated and un-methylated cytosine in DNA. **(A)** The indicated concentrations of Fpg protein [P] were incubated with DNA substrate (50 nM; **Figure 1A,** see **Supplementary Table 3**) at 37°C for 30 min (final volume, 20 µL; **Figure 1B–G**). Fpg (**A–H**, 500 nM; **I** and **J**, 10 nM; K, 5 nM) was incubated with increasing concentration of DNA (**Figure 1A**) containing either **(B)** C**∙**C, **(C)** m*^N^*^4^C**∙**C, **(D)** m^5^C**∙**C, **(E)** m^5^C**∙**T, **(F)** m*^N^*^4,5^C**∙**C, **(G)** C**∙**C, **(H)** T**∙**T, **(I)** dHT**∙**A, **(J)** Tg**∙**A or **(K)** oxo^8^G**∙**C as described in **A**. Each value represents the average (± SD) of 4–13 independent experiments. See **Figure 1A** for explanation of substrate DNA colors.

**Supplementary Figure 4.** Fpg-mediated incision of the C**∙**C mismatch forms no ds break in DNA. Non-denaturing and denaturing PAGE of the DNA substrate with one C**∙**C mismatch (upper panel) were performed following treatment with Fpg. The DNA (1 pmol) was incubated alone or with Fpg (13 pmol) under exactly the same conditions as described previously (see **Figure 1** and Materials and Methods). The incision product was separated from un-incised DNA by non-denaturing (200 V for 1 h) and denaturing (200 V for 2 h) PAGE (see **Figure 1B–D**). The single incision at C on the labeled strand, as was observed carried out by Fpg (shown by unbroken arrows), results in an incision product indistinguishable from substrate DNA (left panel), as monitored by non-denaturing PAGE (green square), and as opposed to denaturing PAGE (brown square). A putative ds break product (left panel) would form if Fpg targeted the mismatched C on the complementary strand following that on the forward strand (shown by broken arrows), and should be viewed as a 39-nt band following non-denaturing PAGE. Such a band was not observed (green square; between the green box drawings) in any of the five independent experiments (ten gel runs) performed. The 5´-label and strand are indicated in magenta; see **Figure 1A** for explanation of other colors.

**Supplementary Figure 5.** Tentative accommodation of the C∙C mismatch in the *E. coli* Fpg active site. This working model is based on crystal structures of Fpg orthologs with certain damaged base residues, as presented in the discussion section. Residues Thr214, Thr215, Leu216 and Lys217 are part of the flexible αF-β9 loop (grey arc), these possibly forming H-bonds (grey broken lines) to the flipped-out substrate C base. Arg108 forms H-bonds to the opposite C base. The Fpg active site was manually inspected and visualised using PyMOL (The PyMOL Molecular Graphics System, Version 2.4 Schrödinger, LLC). Abbreviation: dRP, deoxyribose phosphate.

**Supplementary Figure 6.** Origin and biological consequences of the C∙C DNA mismatch in *E. coli*. This working model summarizes the expected origin of a C∙C mismatch in *E. coli* DNA and its putative destiny. Pol III replicates the genome with high processivity (step 1) and fidelity due to binding to the replication clamp and its 3´→ 5´ exonuclease (exo) function respectively, thus removing all base mismatches efficiently (steps 2 and 3). A C∙C mismatch that evades these defenses might be extended by Pol III (step 4). This is, however, very unlikely. Instead, Pol III leaves the replication clamp for a trans-lesion synthesis (TLS) Pol, being able to synthesize downstream of the lesion, where Pol IV is, in this case, most likely (step 5). Two scenarios are possible after Pol IV departure. Either the C∙C mismatch survives the ongoing round of replication and Pol III replicates both strands, this resulting in a G∙C → C∙G mutation in 50% of the offspring (step 6a and b), or Fpg is recruited to C∙C and initiates BER (step 7). In the latter case we propose that the opposed Cs are selected randomly, yielding a 50% chance of mutagenesis (step 7a and b) as in the first scenario. Innocuous reactions or events are shown in blue, in red if aberrant or causing mutagenesis, short arrows showing class switch between replicative and TLS Pol or between Pol and Fpg (🠝, leaves DNA; 🠟, recruited to DNA).

**Supplementary Figure 7.** Comparison of velocity data of steady-state and rapid equilibrium approximations and v_pfo_, S_pfo_ expressions with corresponding numerical calculation (see Kinetic and Computational Methods). Initial substrate concentration in all calculations is [S]_0_ = 5 x 10^–9^ M and initial enzyme concentration [E]_0_ = [E]_tot_ = 500 x 10^–9^ M. Velocities are shown as semi-logarithmic plots. The linearity in the plots shows that the velocities decrease exponentially. Symbols with 'num' subscripts refer to the numerical results, while 'ss' and 're' subscripts refer to steady-state and rapid equilibrium values, respectively. Panels **(A)**, **(B)**, **(C)** and **(D)** refer to the rate constant combinations 1, 6, 7, 8 (**Supplementary Table 2**), respectively. The remaining four rate constant combinations 2, 3, 4 and 5 are shown in **Supplementary Figure 8**.

**Supplementary Figure 8.** Comparison of velocity data of steady-state and rapid equilibrium approximations, and v_pfo_ and S_pfo_ expressions with corresponding numerical calculation (see Kinetic and Computational Methods). Initial substrate concentration in all calculations is [S]_0_ = 5 x 10^–9^ M and initial enzyme concentration [E]_0_ = [E]_tot_ = 500 x 10^–9^ M. Velocities are shown as semi-logarithmic plots. The linearity in the plots shows that the velocities decrease exponentially. Symbols with 'num' subscripts refer to the numerical results, while 'ss' and 're' subscripts refer to steady-state and rapid equilibrium values, respectively. Panels **(A)**, **(B)**, **(C)** and **(D)** refer to the rate constant combinations 2, 3, 4 and 5 (**Supplementary Table 2**), respectively. The remaining four rate constant combinations 1, 6, 7, 8 are shown in **Supplementary Figure 7**.


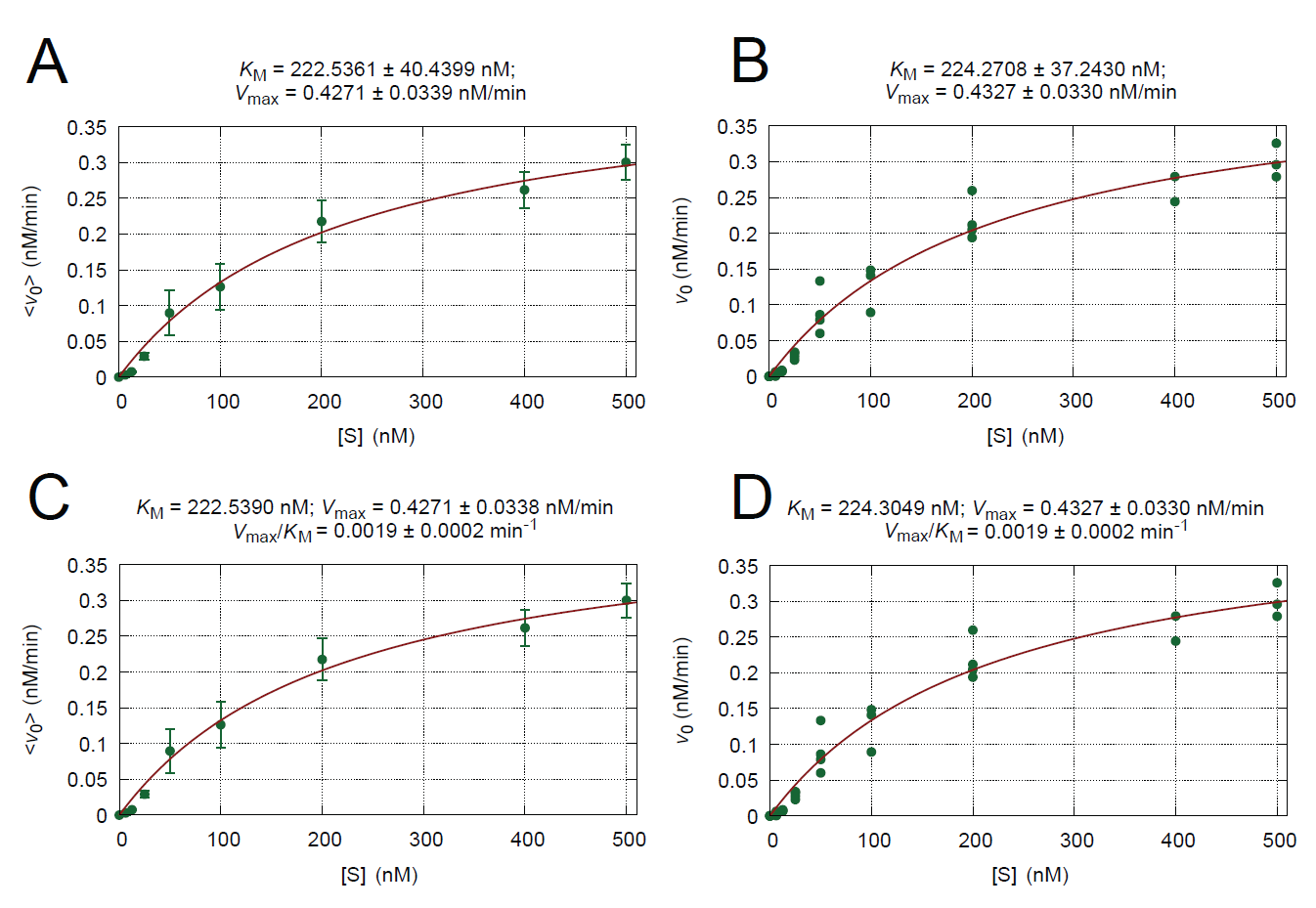


**Supplementary Figure 9.** Curve fits of the C**∙**C data. Left panels **(A** and **C)** show the curve fits based on the average velocity data, while right panels **(B** and **D)** show the curve fits based on all individual data. Upper panels **(A** and **B)** show the fit of *V*_max_ and *K*_M_ to *v*_0_ = $\frac{V\text{max } \left[ S \right]}{K\text{M} + \left[ S \right]}$. Lower panels **(C** and **D)** show the fit of $\alpha$ = *V*_max_/*K*_M_ and $\beta$ = *K*_M_ to the equation (Johnson, 2019) *v*_0_ = $\frac{\alpha\cdot[S]}{1+\frac{\alpha\cdot[S]}{\beta}}$ using the average velocities (panels **C**) and all velocities (panels **D**). The values reported in **Table 1** are from panels B and D, but for comparison the curve fits on the average data are also included.


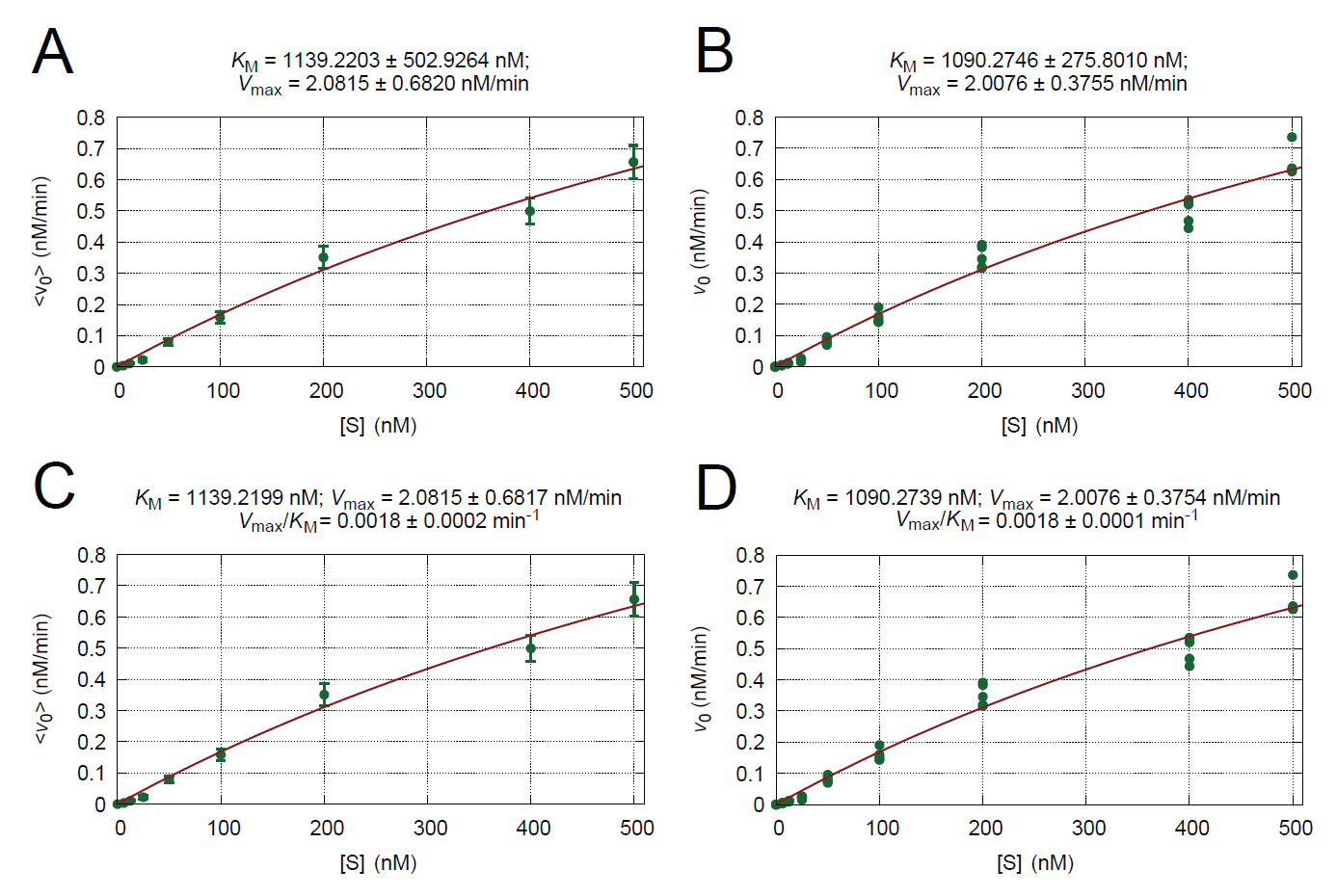


**Supplementary Figure 10.** Curve fits of the C**∙**C data. See legend to **Supplementary Figure 9** for explanations.


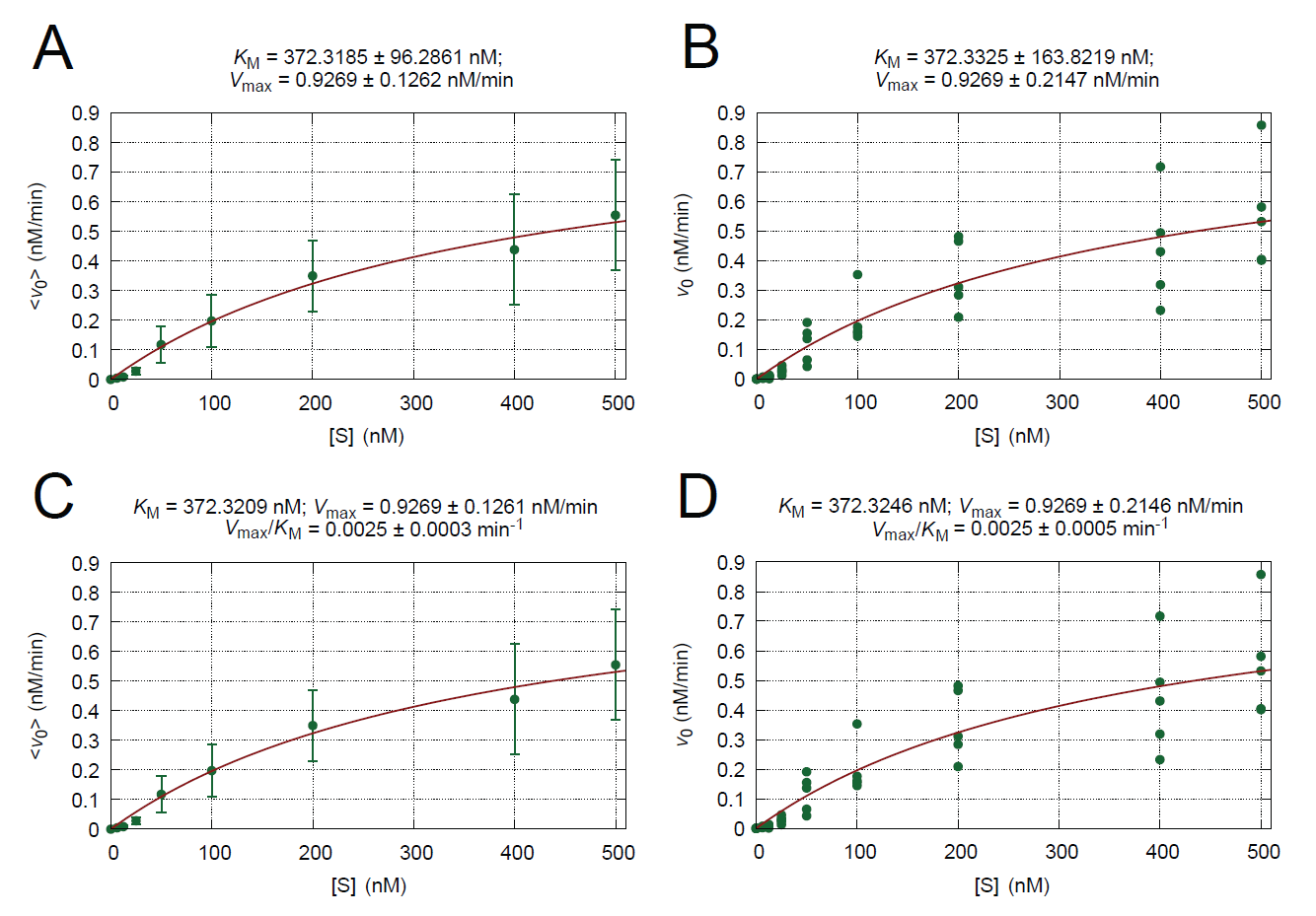


**Supplementary Figure 11.** Curve fits of the T**∙**T data. See legend to **Supplementary Figure 9** for explanations.


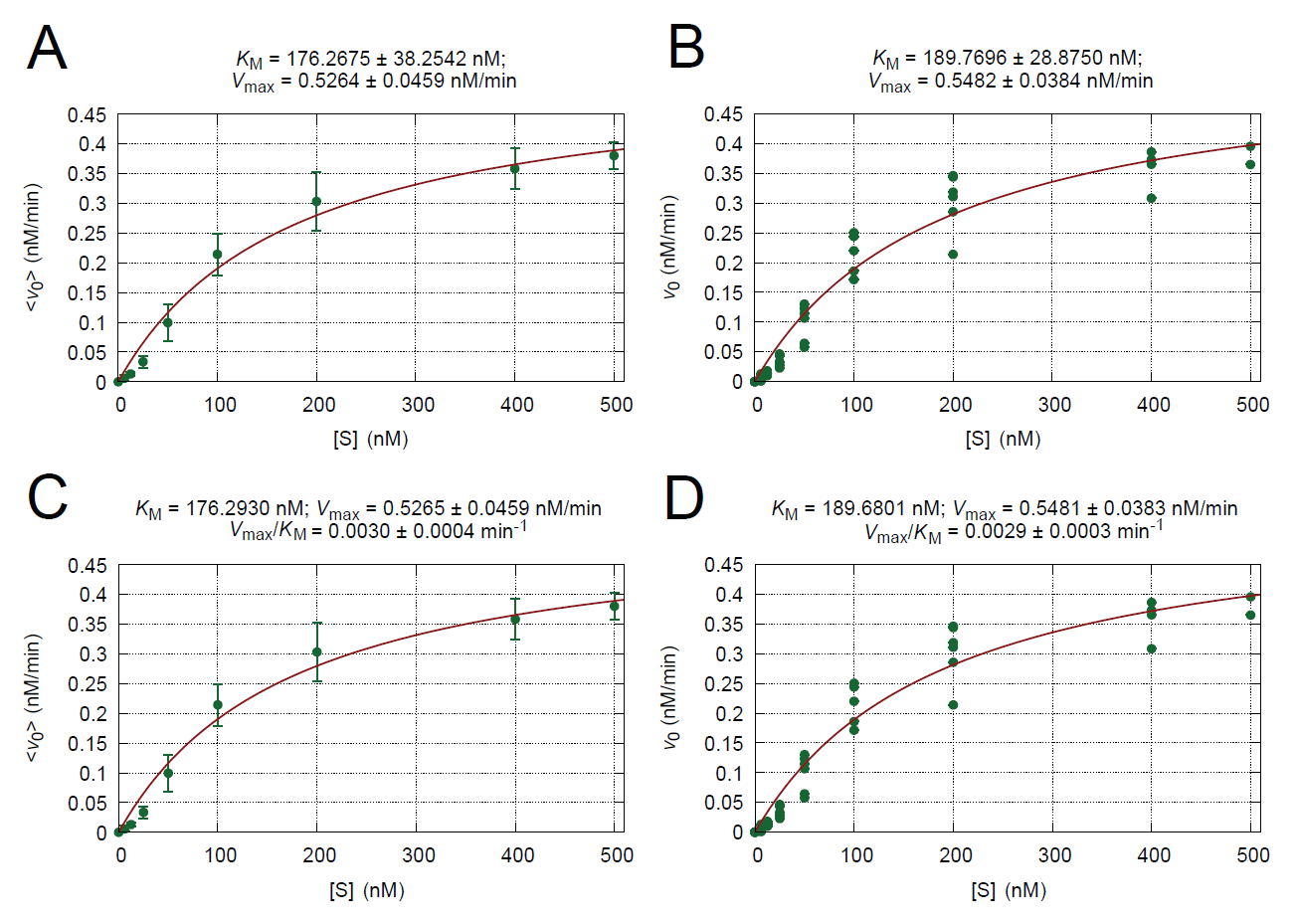


**Supplementary Figure 12.** Curve fits of the m*^N^*^4^C**∙**C data. See legend to **Supplementary Figure 9** for explanations.


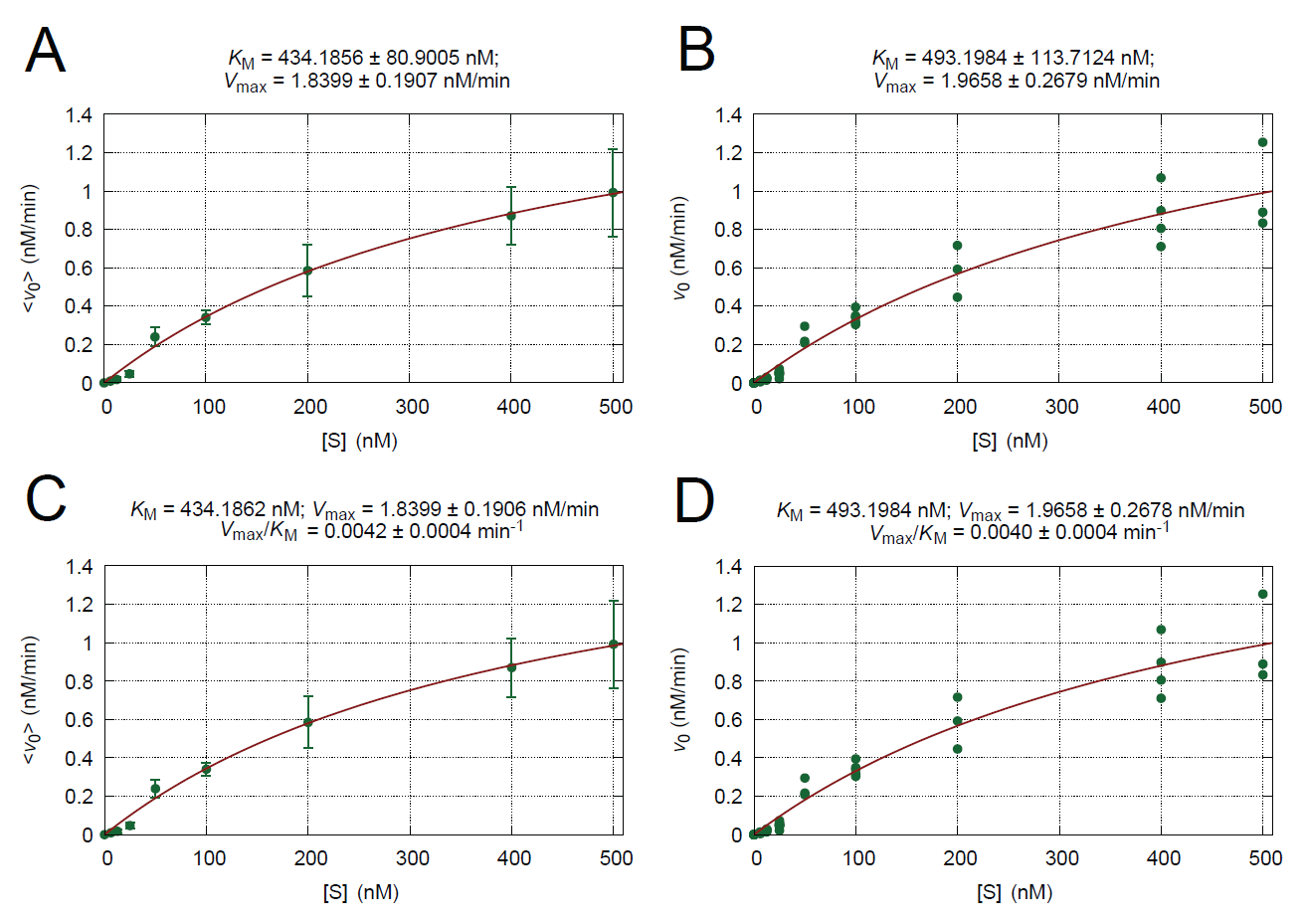


**Supplementary Figure 13.** Curve fits of the m^5^C**∙**C data. See legend to **Supplementary Figure 9** for explanations.


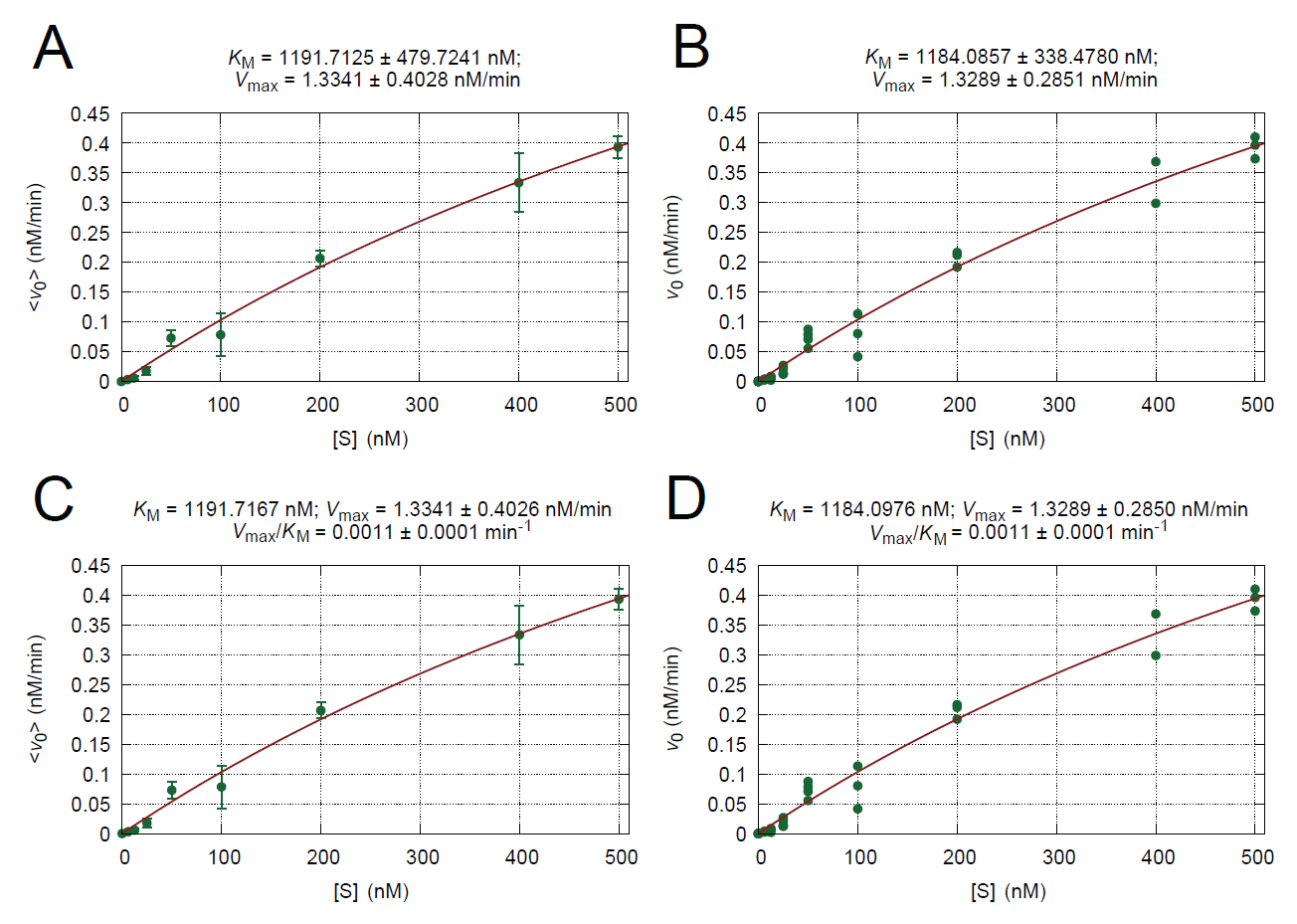


**Supplementary Figure 14.** Curve fits of the m^5^C**∙**T data. See legend to **Supplementary Figure 9** for explanations.


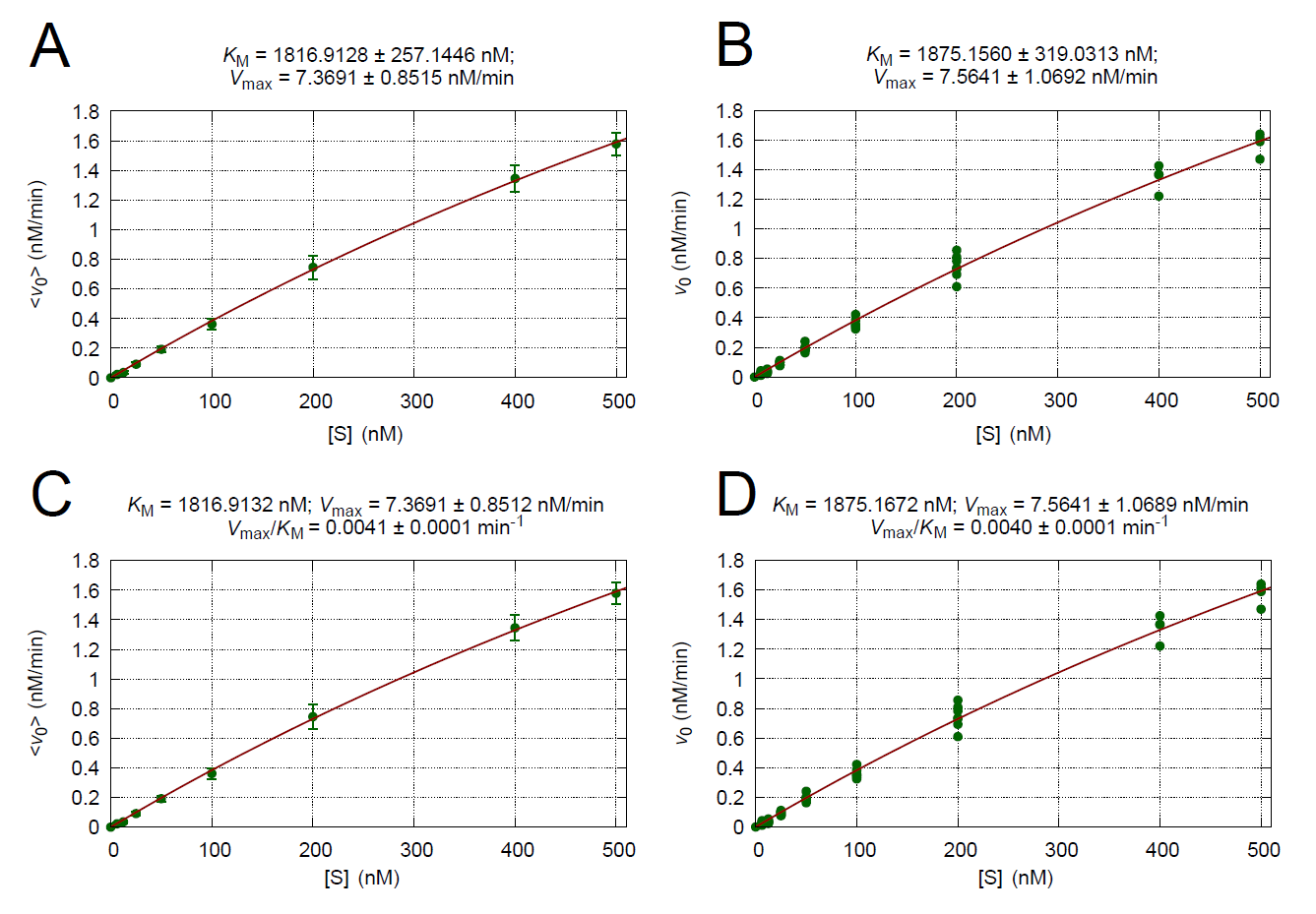


**Supplementary Figure 15.** Curve fits of the m*^N^*^4,5^C**∙**C data. See legend to **Supplementary Figure 9** for explanations.


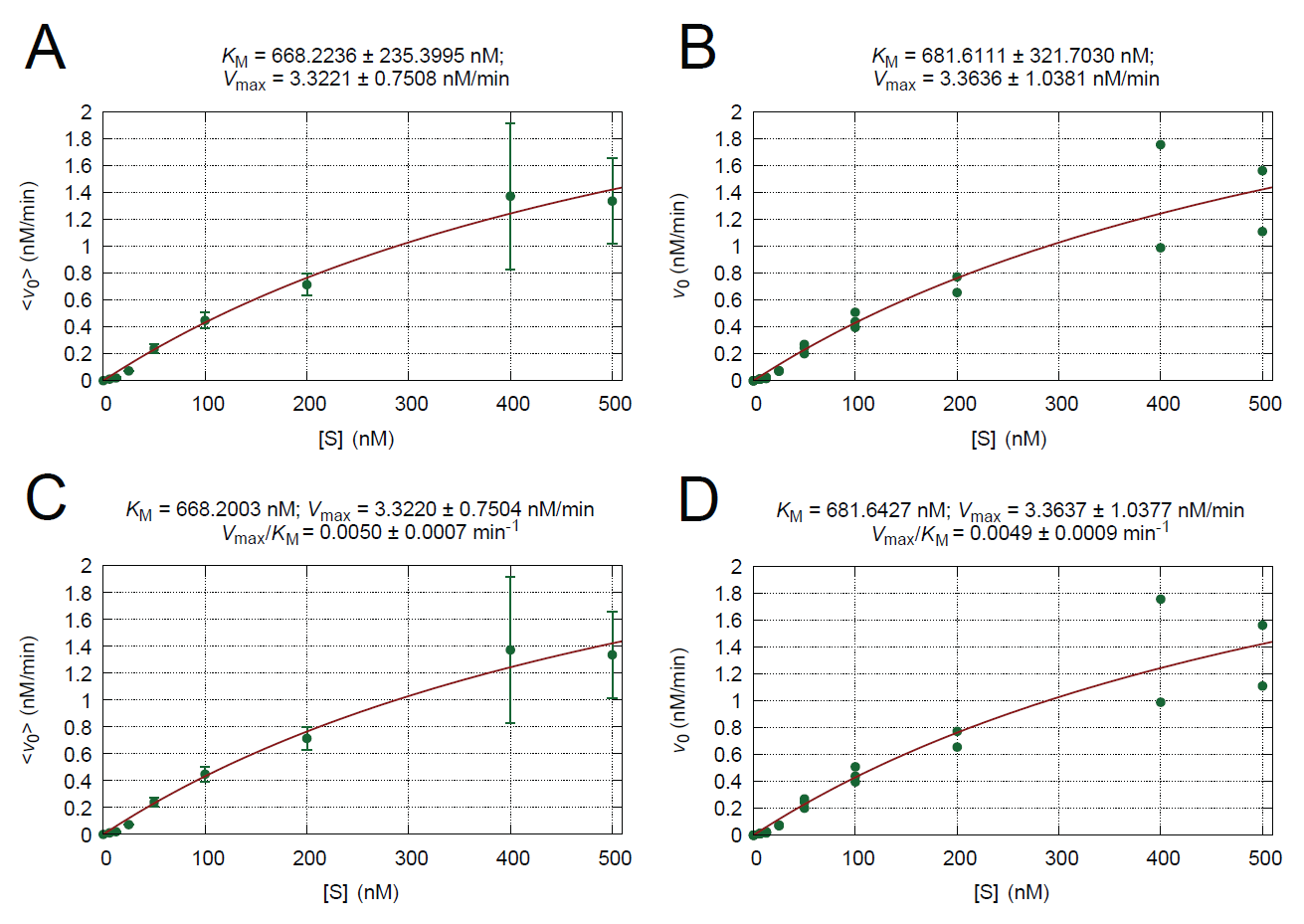


**Supplementary Figure 16.** Curve fits of the dHT**∙**A data. See legend to **Supplementary Figure 9** for explanations.


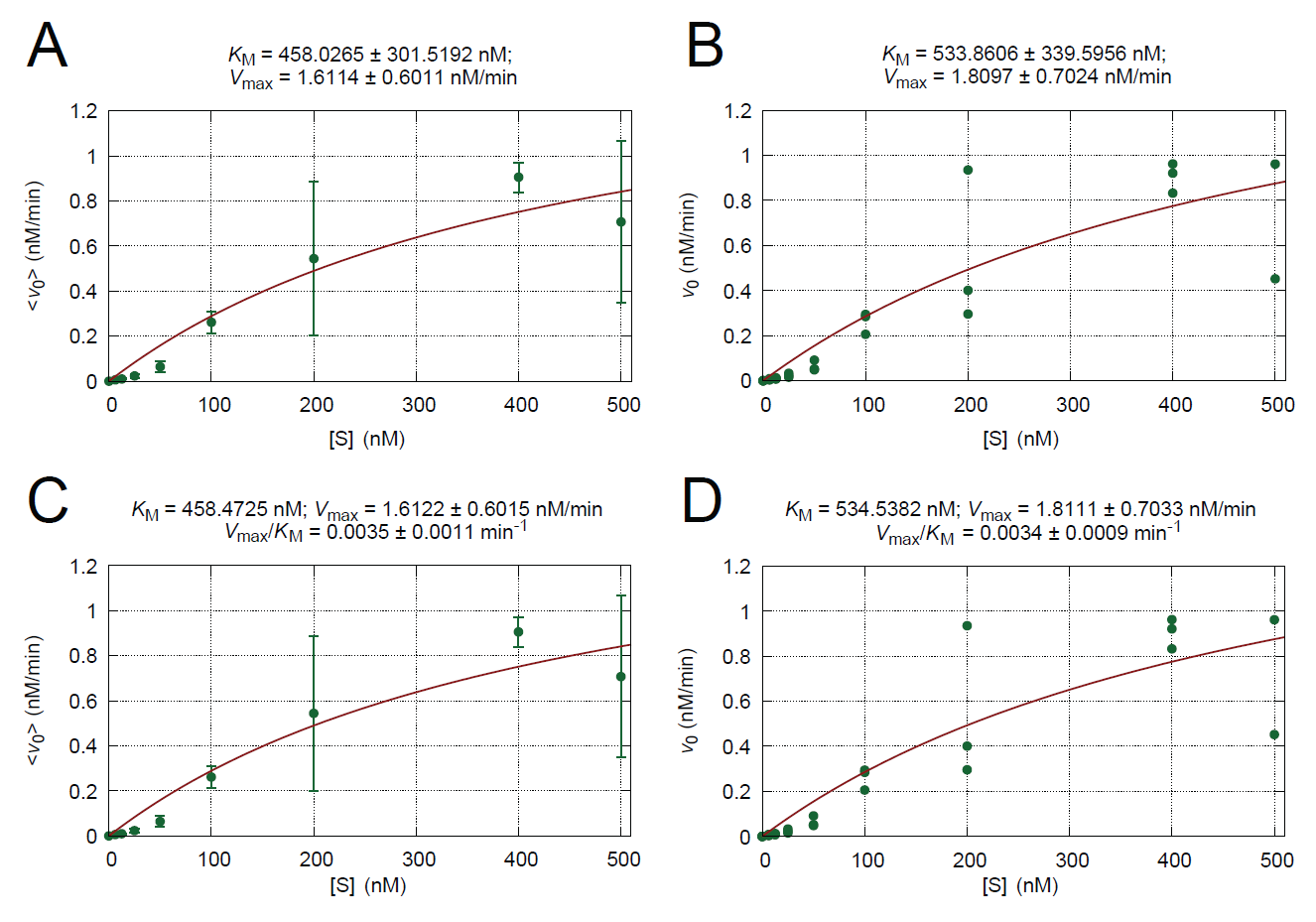


**Supplementary Figure 17.** Curve fits of the Tg**∙**A data. See legend to **Supplementary Figure 9** for explanations.


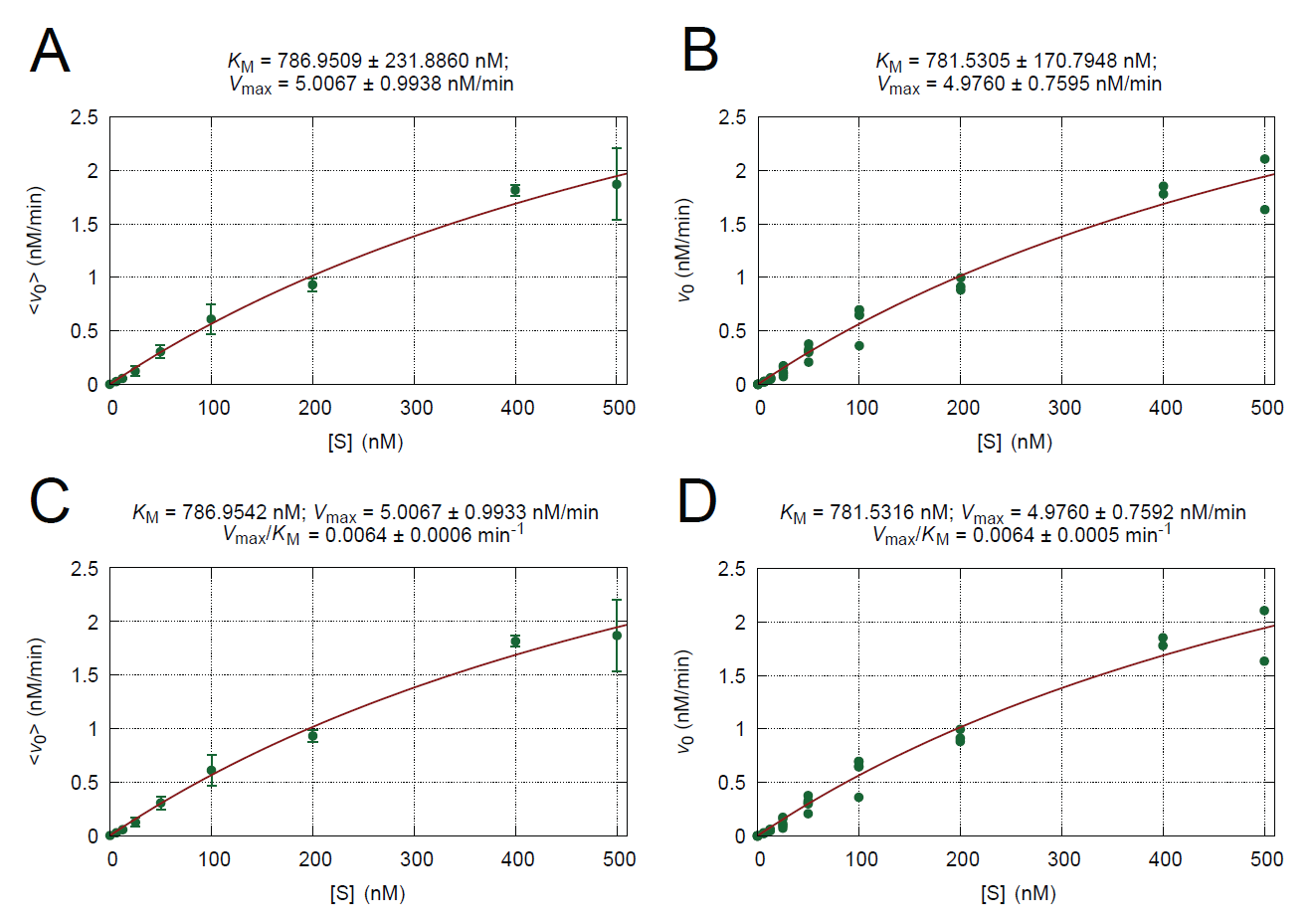


**Supplementary Figure 18.** Curve fits of the oxo^8^G**∙**C data. See legend to **Supplementary Figure 9** for explanations.

## Supplementary Tables

**Supplementary Table 1.** MS analysis of the commercial (New England Biolabs) Fpg preparation used in this study.

**Supplementary Table 2.** Rate constant combinations used in testing the steady-state and rapid equilibrium approximations.

| Combination  No.* | 1 | 2 | 3 | 4 | 5 | 6 | 7 | 8 |
| --- | --- | --- | --- | --- | --- | --- | --- | --- |
| *k*_1_, M^−1^ min^−1^ | 10^10^ | 10^10^ | 10^10^ | 10^7^ | 10^7^ | 10^10^ | 10^7^ | 10^7^ |
| *k*_−1_, min^−1^ | 10^6^ | 10^6^ | 10^2^ | 10^6^ | 10^2^ | 10^2^ | 10^6^ | 10^2^ |
| *k*_2_, min^−1^ | 10^7^ | 50 | 10^7^ | 10^7^ | 10^7^ | 50 | 50 | 50 |

*orange cells indicate upper range values; blue cells indicate lower range values.

**Supplementary Table 3.** Pseudo-First-Order Kinetics.


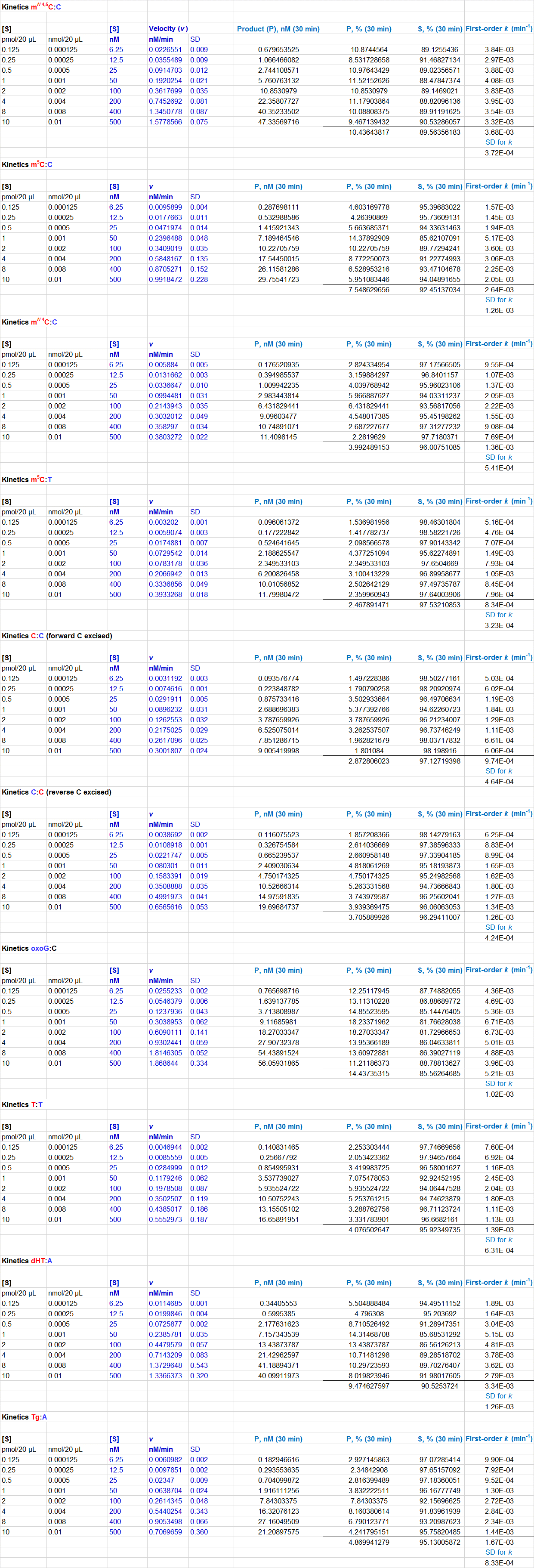


**Supplementary Table 4.** Fpg-mediated DNA incision at different enzyme concentrations and time. See **Supplementary Figure 3A**.
